# TaHsfA6f is a transcriptional activator that regulates a suite of heat stress protection genes in wheat (*Triticum aestivum* L.) including previously unknown Hsf targets

**DOI:** 10.1093/jxb/eru462

**Published:** 2014-11-26

**Authors:** Gang-Ping Xue, Janneke Drenth, C. Lynne McIntyre

**Affiliations:** CSIRO Plant Industry, 306 Carmody Road, St Lucia, Qld 4067, Australia

**Keywords:** Gene regulation, Golgi anti-apoptotic protein, heat shock factor, Rubisco activase, transcriptional activator, wheat.

## Abstract

Heat stress is a significant environmental factor adversely affecting crop yield. Crop adaptation to high-temperature environments requires transcriptional reprogramming of a suite of genes involved in heat stress protection. This study investigated the role of TaHsfA6f, a member of the A6 subclass of heat shock transcription factors, in the regulation of heat stress protection genes in *Triticum aestivum* (bread wheat), a poorly understood phenomenon in this crop species. Expression analysis showed that *TaHsfA6f* was expressed constitutively in green organs but was markedly up-regulated during heat stress. Overexpression of *TaHsfA6f* in transgenic wheat using a drought-inducible promoter resulted in up-regulation of heat shock proteins (HSPs) and a number of other heat stress protection genes that included some previously unknown Hsf target genes such as Golgi anti-apoptotic protein (GAAP) and the large isoform of Rubisco activase. Transgenic wheat plants overexpressing *TaHsfA6f* showed improved thermotolerance. Transactivation assays showed that TaHsfA6f activated the expression of reporter genes driven by the promoters of several HSP genes (*TaHSP16.8*, *TaHSP17*, *TaHSP17.3*, and *TaHSP90.1-A1*) as well as *TaGAAP* and *TaRof1* (a co-chaperone) under non-stress conditions. DNA binding analysis revealed the presence of high-affinity TaHsfA6f-binding heat shock element-like motifs in the promoters of these six genes. Promoter truncation and mutagenesis analyses identified TaHsfA6f-binding elements that were responsible for transactivation of *TaHSP90.1-A1* and *TaGAAP* by TaHsfA6f. These data suggest that TaHsfA6f is a transcriptional activator that directly regulates *TaHSP*, *TaGAAP*, and *TaRof1* genes in wheat and its gene regulatory network has a positive impact on thermotolerance.

## Summary

A wheat HsfA6 member acts as a transcriptional activator for up-regulation of a suite of heat stress protection genes including previously unknown Hsf targets such as Golgi anti-apoptotic protein.

## Introduction

Heat stress is one of the major environmental factors that have a negative impact on crop yields. Heat stress causes inactivation of many thermo-labile proteins, accumulation of harmful reactive oxygen species in plant cells, and in severe cases induces programmed cell death (Xue and McIntyre, 2011; Mittler *et al.*, 2012; Grover *et al.*, 2013). Heat stress also rapidly induces a suite of heat stress protection genes, such as those encoding heat shock proteins (HSPs), to very high levels (Kotak *et al.*, 2007*a*; Mittler *et al.*, 2012; Sarkar *et al.*, 2014; Xue *et al.*, 2014). Genotypic variation in thermotolerance in wheat is linked to the levels of HSP transcripts and proteins (Vierling and Nguyen, 1992; Joshi *et al.*, 1997; Skylas *et al.*, 2002). Many HSP proteins are known to act as molecular chaperones for the protection of thermo-labile proteins against heat-induced denaturation in plant cells (Wang *et al.*, 2004; Basha *et al.*, 2010; Waters, 2013).

Heat shock factors (Hsfs) are transcription factors and are present in all eukaryotic organisms. In plants, Hsf proteins form a large family, with 21 members in *Arabidopsis thaliana*, 25 members in rice (*Oryza sativa* L), and at least 56 members in wheat (Scharf *et al.*, 2012; Xue *et al.*, 2014). Hsf proteins in plants are divided into three classes: HsfA, HsfB, and HsfC (Scharf *et al.*, 2012). Several HsfA subclasses (A1, A2, A3, A4, and A9) have been shown to serve as transcriptional activators for HSP genes (Mishra *et al.*, 2002; Charng *et al.*, 2007; Schramm *et al.*, 2008; Pérez-Salamó *et al.*, 2014; Kotak *et al.*, 2007*b*; Xue *et al.*, 2014), whereas HsfA5 acts as a specific repressor for HsfA4 (Baniwal *et al.*, 2007). Hsf proteins contain a DNA-binding domain and bind to heat shock elements (HSEs) with a consensus sequence of GAAnnTTCnnGAA (Santoro *et al.*, 1998; Nover *et al.*, 2001; Guo *et al.*, 2008; Mittal *et al.*, 2011; Xue *et al.*, 2014). Although many HsfA proteins are capable of binding to this typical HSE sequence, each subclass of HsfA proteins is known to regulate a subset of heat stress-responsive genes (Busch *et al.*, 2005; Nishizawa *et al.*, 2006; Yokotani *et al.*, 2008; Liu and Charng, 2013). HSEs are present in the promoters of many HSP genes (Nover *et al.*, 2001; Guo *et al.*, 2008; Mittal *et al.*, 2011; Xue *et al.*, 2014). Hsf proteins have been proposed to play a key role in regulating the expression of HSP genes, which have a significant impact on thermotolerance (Kotak *et al.*, 2007*a*; Scharf *et al.*, 2012).

In addition to the importance of HSPs in the protection of plant cells during heat stress, other classes of proteins are also known to be important for the adaptation of plants to heat stress. These include co-chaperones [e.g. Rof1 (FKBP62) (Meiri and Breiman, 2009)], enzymes involved in the synthetic pathways of raffinose family oligosaccharides [e.g. galactinol synthase (Panikulangara *et al.*, 2004)], and enzymes for the protection of cells from damage by reactive oxygen species [e.g. ascorbate peroxidase (Panchuk *et al.*, 2002)]. Heat stress is also known to adversely affect carbon assimilation through inactivation of some thermo-labile proteins involved in photosynthesis. One well-known example of these proteins is Rubisco activase, which is required for maintaining Rubisco in active form (Portis, 2003). Rubisco activity and CO_2_ exchange rate in wheat leaves decrease during heat stress (Law and Crafts-Brandner, 1999).

Severe heat stress can also lead to programmed cell death (Mittler *et al.*, 2012). A number of anti-apoptotic proteins are known to have an important role in suppressing programmed cell death in eukaryotes (Watanabe and Lam, 2009; Ishikawa *et al*, 2011). These proteins include Bax (pro-apoptotic protein) inhibitor 1 (BI-1) proteins, Golgi anti-apoptotic proteins (GAAP), and inhibitor of apoptosis proteins (IAPs) (Carrara *et al.*, 2012; Marivin *et al.*, 2012). Both BI-1 and GAAP proteins are highly hydrophobic and contain the transmembrane Bax inhibitor-containing motif. IAP family proteins contain a baculoviral IAP repeat domain. BI-1 in *Arabidopsis* is known to play a positive role in suppressing the programmed cell death induced by biotic and abiotic stress including heat shock (Watanabe and Lam, 2006). Loss-of-function mutants of BI-1 in *Arabidopsis* exhibit increased sensitivity to heat shock-induced cell death (Watanabe and Lam, 2006). These authors have also shown that expression of *AtBI1* mRNA was up-regulated in *Arabidopsis* leaves by heat in early hours of heat treatment and before the activation of cell death. Transgenic tomato plant overexpressing an anti-apoptotic gene from the IAP family enhances thermotolerance (Li *et al.*, 2010). However, transcription factors that directly regulate expression of anti-apoptotic proteins in plants during heat stress have not been reported to date.

Some Hsf genes are constitutively expressed in plants. In particular, constitutively expressed subclass *HsfA1* genes serve as master regulators for triggering heat response (Mishra *et al.*, 2002; Liu *et al.*, 2011; Liu and Charng, 2012). In wheat, most HsfA genes are expressed at moderately high levels under normal conditions (Xue *et al.*, 2014). It is generally considered that constitutively expressed HsfA1 proteins are in inactive forms through their interaction with some repressors under normal conditions (Scharf *et al.*, 2012). HsfA1a in tomato has been shown to maintain its inactive monomer state by association with HSP90/HSP70 under non-heat stress conditions (Hahn *et al.*, 2011). However, a number of transgenic studies have shown that constitutive overexpression of some HsfA members can up-regulate a subset of heat inducible genes and enhance thermotolerance in plants (Prändl *et al.*, 1998; Li *et al*, 2005; Nishizawa *et al.*, 2006; Ogawa *et al.*, 2007; Yokotani *et al.*, 2008; Liu and Charng, 2013), as overexpression of a Hsf is likely to make Hsf in excess of its repressor.

In this study, the role of a subclass HsfA6 member of the wheat Hsf family (TaHsfA6f) in the adaptation of wheat to heat stress was investigated. Functional studies on the role of subclass HsfA6 members in plant adaptation to heat stress have not been reported in any plant species. To understand the biological function of TaHsfA6f, this study focused on the identification of target genes regulated by TaHsfA6 in wheat. Transgenic wheat lines overexpressing *TaHsfA6f* driven by a drought-inducible promoter were generated. Affymetrix array analysis revealed that a large number of HSP genes and other classes of heat stress protection genes were up-regulated in the *TaHsfA6f* overexpressing lines. In particular, an anti-apoptotic gene (*TaGAAP*) and a Rubisco activase large isoform (*TaRCA-L*) were also identified as TaHsfA6f target genes. TaHsfA6f target genes also include a C4-type zinc finger transcription factor (*TaC4ZFP*). These three genes are previously unknown Hsf targets. Analysis of selected up-regulated genes by quantitative RT-PCR revealed that all of these genes were markedly induced in wheat leaves during heat stress. High affinity TaHsfA6f-binding HSEs were found in the promoters of many TaHsfA6f up-regulated genes examined. Transactivation analysis showed that TaHsfA6f served as a transcriptional activator capable of activating the expression of reporter genes driven by the promoters of *TaGAAP* and *TaRof1* in addition to HSP genes. These observations demonstrate that a HsfA6 transcription factor plays a role in regulation of several classes of heat stress protection genes.

## Materials and methods

### Plant materials and growth conditions

*T. aestivum*. (cv. Bobwhite) plants were grown in a controlled-environment growth room in 1.5-l pots, containing a 3:1:1 mix of sand:soil:peat under night/day conditions of 16-h light (500 µmol m^–2^s^–1^), 16/20 °C, and 80/60% relative humidity. Heat treatment of one-month-old plants at 36 °C commenced at 2h after lights on and the leaves and roots of heat-treated plants were harvested after 1.5h and 5h of heat treatment. Some plants also went through a 3-day heat treatment regime with 5h at 36 °C per day. Control plants grown at 20 °C were harvested at the same time as for the 1.5-h heat treated samples.

### Isolation of total RNA and *TaHsfA6f* cDNA

Total RNA was isolated from wheat samples using Plant RNA Reagent (Invitrogen, California, USA), according to the manufacturer’s instructions. RNA was further purified through a Qiagen RNeasy column (Qiagen, Australia) after pre-treatment with RNase-free DNase I (Xue and Loveridge, 2004).

*TaHsfA6f* cDNA was isolated from the leaves of heat-treated wheat plants using 3ʹ-RACE with primers designed from the partial *TaHsfA6f* cDNA containing the N-terminal sequence. The PCR-amplified product was cloned into pGEM-T Easy vector (Promega) and sequenced using a BigDye terminator cycle sequencing kit (Applied Biosystems, Foster City, USA). The *TaHsfA6f* cDNA sequence was deposited in GenBank (KJ774108).

### Plasmid construction

The *HVA1s* promoter-driven *TaHsfA6f* construct (barley-HVA1s:TaHsfA6f:rice-rbcS3’) was made by inserting the coding region of *TaHsfA6f* after the barley *HVA1s* promoter, using a pHVA1sGUSR plasmid as reported by Xiao and Xue (2001). pTaHsfA6f–CELD was constructed by translational fusion of the coding region sequence of *TaHsfA6f* to the N-terminus of the 6×His-tagged CELD reporter (Xue, 2005). *CelD* encodes a 1,4-β-glucanase (cellulase) (Xue *et al.*, 1992). The CELD-positive clones containing in-frame fusion of TaHsfA6f–CELD were identified using a CM-cellulose plate (Xue *et al.*, 2003). Maize *Ubi1* promoter-driven *TaHsfA6f*, *TaHsfA1b*, and *TaHsfA4e* effector constructs (maize-Ubi1:TaHsfA:rice-rbcS3ʹ) were constructed by replacing *xylanase* in pUbiSXR (Vickers *et al.*, 2003) with the coding region of a *TaHsfA* cDNA. *TaHSP16.8*, *TaHSP17*, *TaHSP17.3 TaHSP90.1-A1*, *TaGAAP*, *TaRof1*, and *TaRCA-L* promoter-driven *GFP* reporters were constructed by replacing the *HVA1s* promoter in a HVA1s:GFP construct (Xue and Loveridge, 2004) with the PCR-amplified fragment of the promoters of interest (Xue *et al.*, 2014; see the HSP promoter isolation section below). *TaHSP90.1-A1* promoter mutants (psHSP90gfp, pΔHSE90gfp, and pHSE90gfp) were constructed previously (Xue *et al.*, 2014). HSE90-miniDhn6gfp construct was made by adding three repeats of TaHSP90.1E1 to the upstream of a minimal promoter (PminiDhn6), which was derived from a drought-inducible barley *Dhn6* gene and was inactive by itself (Xue, 2003). A *TaGAAP* promoter-driven xylanase (*xynA*) reporter gene was constructed by replacing the *Ubi1* promoter in pUbiSXR (Vickers *et al.*, 2003) with the PCR-amplified fragment of the *TaGAAP* promoter. Truncated and HSE mutant constructs of *TaGAAP* promoter were made using PCR-based promoter truncation and site-directed mutagenesis.

### Production of transgenic wheat overexpressing *TaHsfA6f*

The *HVA1s* promoter-driven *TaHsfA6f* construct (barley-HVA1s:TaHsfA6f:rice-rbcS3ʹ) and the selectable marker cassette containing rice-act1:bar:nos3ʹ (Xue *et al.*, 2011*b*) were used to co-transform Bobwhite wheat plants using the particle bombardment as described by Pellegrineschi *et al.* (2002). Transgenic plants were selected with the herbicide phosphinothricin and grown in a controlled environment growth room as described above. The presence of the barley-HVA1s:TaHsfA6f:rice-rbcS3’ cassette was verified by real-time PCR using genomic DNA (Kooiker *et al.*, 2013).

### Expression analysis using Affymetrix wheat genome array

The Affymetrix wheat genome array contains 61 127 probe sets representing 55 052 transcripts from genes distributed across all 42 chromosomes in the wheat genome. One-week-old seedlings of TaHsfA6f transgenic and wild-type Bobwhite plants were treated with 15% PEG (MW 8000) in a controlled environment growth room at 16 °C/20 °C (night/day) for 3 d to induce the expression of the *HVA1s* promoter-driven *TaHsfA6f* transgene. RNA from whole seedlings was extracted and processed as described above. Affymetrix wheat genome array expression profiling was performed as described by Xue *et al.* (2013). The Affymetrix array data were normalized using GeneChip robust multiarray average (Wu *et al.* 2004). Normalized values were converted to non-log values for comparative analysis of gene expression between transgenic and wild-type plants.

### Expression analysis using quantitative RT-PCR

The mRNA levels of the genes of interest were quantified from cDNA samples synthesized from DNase I-treated total RNA using real-time PCR with a ViiA^TM^ 7 system (Applied Biosystems) and SYBR Green PCR Master Mix (Applied Biosystems) according to the manufacturer’s instructions. The gene-specific primers were designed at the C-terminal coding or 3ʹ-untranslated region. The sequences of real-time PCR primer pairs are listed in Supplementary Table S1.

Two wheat housekeeping genes (*TaRPII36* and *TaRP15*) were selected as internal reference genes for the calculation of relative transcript levels of the genes of interest (Xue *et al.*, 2006, 2008*a*). The mRNA levels of these internal reference genes were similar in the control and heat–treated samples and checked by the use of an external reference mRNA synthesized from a bovine cDNA (Xue *et al.*, 2011*a*). The PCR efficiency of each primer pair was determined by a dilution series of samples. The specificity of real-time PCR amplification was confirmed by a single peak in melting temperature curve analysis of real-time PCR-amplified products. Relative quantitation of mRNA levels was as described by Shaw *et al.* (2009). The apparent expression level of each gene relative to an internal reference gene (*TaRP15*) was calculated as described by Stephenson *et al.* (2007).

### DNA-binding activity assays

TaHsfA6f–CELD fusion protein tagged with 6×His was purified using Ni-NTA magnetic agarose beads (Xue, 2005). Biotin-labelled double-stranded oligonucleotide probes containing HSE-like sequences were synthesized by filling in partially double-stranded oligonucleotides using *Taq* polymerase reaction as described previously (Xue *et al.*, 2006). HSE-like sequences were derived from the promoters of the following TaHsfA6f-up-regulated genes: *TaGAAP* (GenBank accession number: KJ685918), *TaRCA-L* (KJ685916), *TaRof1* (KJ685917), *TaHSP16.8* (KJ685920), *TaHSP16.9b* (Supplementary Figure S1), *TaHSP1*7 (KF208539), *TaHSP17.3* (KJ685919), *TaHSP62.4* (Supplementary Figure S1), *TaHSP90.1-A1* (KF208540), and *TaHSP101* (Supplementary Figure S1).

The measurement of DNA-binding activity of TaHsfA6f–CELD was performed as described by Xue (2002) using StreptaWell High Bind (streptavidin-coated 96-well plates from Roche) and binding/washing buffer (25mM HEPES/KOH, pH 7.0, 50mM KCl, 2mM MgCl_2_, and 0.5mM DTT) containing 0.15 µg µl^–1^ shared herring sperm DNA, 0.3mg ml^–1^ bovine serum albumin, 10% glycerol, and 0.025% Nonidet P-40. About 20 000 fluorescent units h^–1^ of the CELD activity of TaHsfA6f–CELD fusion protein and 0.4 pmol of biotinylated probes were used per assay. The cellulase activity of the CELD fusion proteins bound to immobilized biotinylated probes was measured by incubation in 100 µl of the CELD substrate solution (1mM methylumbelliferyl β-d-cellobioside in 50mM Na-citrate buffer, pH 6.0) at 40 °C for 4h. A biotin-labelled double-stranded oligonucleotide without a HSE was used as a control of background activity in DNA-binding assays.

### Transactivation assays

Transactivation of reporter genes by a maize *Ubi1* promoter-driven *TaHsfA* effector construct was analysed as described previously (Xue, 2003). Constructs were introduced into the seedlings of wheat (cv. Bobwhite) by particular bombardment (Xue *et al.*, 2003). An effector gene was co-introduced with a *GFP* or *xynA* reporter gene driven by the promoter of interest to determine the transactivation activity. The reporter genes without a *TaHsfA* effector construct were used as a control. A maize *Ubi1* promoter-driven β-glucuronidase (Ubi1:GUS+) construct (pUbiGUS+, Vickers *et al.*, 2003) was also co-bombarded for validation of transformation events among assays. The bombarded seedlings were kept at room temperature (22 °C) or a heat stress temperature in dark until examination of GFP foci (usually for about 20h except where they are indicated). *GFP* expression was visualized as green GFP foci under a fluorescence microscope. Tissue sections that had GFP foci were subsequently stained for histochemical detection of GUS activity (Jefferson, 1987). When *xynA* was used as a reporter gene, the reporter expression was quantitatively determined by measurement of xylanase activity and Ubi1:GUS+ construct activity (Ubi1:GUS+ used for normalisation of transformation efficiency between assays) (Xue *et al.*, 2011*a*).

### Isolation of promoter sequences

Promoter sequences were isolated using PCR-amplification of genomic DNA of *T. aestivum* genotype SB169 (Xue *et al.*, 2008*b*). PCR primers designed for isolation of the following five promoters were based on assembled sequences through extension of EST or cDNA sequences using the wheat genome sequence database in CerealDB (Wilkinson *et al.*, 2012). The PCR-amplified DNA fragments were cloned and sequenced. The isolated promoter sequences were deposited in GenBank [*TaRCA-L* (1184bp upstream of the translation start codon), KJ685916; *TaRof1* (815bp), KJ685917; *TaGAAP* (1045bp), KJ685918; *TaHSP17.3* (1652bp), KJ685919; *TaHSP16.8* (1253bp), KJ685920].

### Phylogenetic analysis

Phylogenetic analysis was conducted to identify the TaHsfA6f subclass position among TaHsfA members that were reported previously (Xue *et al.*, 2014). Hsf DNA-binding domain and heptad repeat region (HR-A/B) sequences of Hsf proteins were used for generation of a phylogenetic tree by ClustalW alignment and the unrooted neighbor-joining method using MEGA 6.0 (Tamura *et al.*, 2011).

### Thermotolerance test

Five-day-old seedlings of *TaHsfA6f* transgenic lines and Bobwhite were treated with a nutrient solution [0.08% Aquasol (Yates, Australia), 2.5mM CaCl_2_, and 1mM MgCl_2_] containing 15% PEG for 2 d, followed by heat treatment at 45 °C for 2h, and then recovered at 16 °C /20 °C (night/day) in a controlled-environment growth room (normal plant growth conditions were used) for 3 weeks. In the first two days of recovery 15% PEG was included in the nutrient solution for maintaining *TaHsfA6f* transgene expression. The shoot length was measured after the end of the recovery phase. Conditions for other control experiments are specified in the figure legends.

## Results

### *TaHsfA6f* is constitutively expressed in various organs and up-regulated by heat stress

**Fig. 1.**
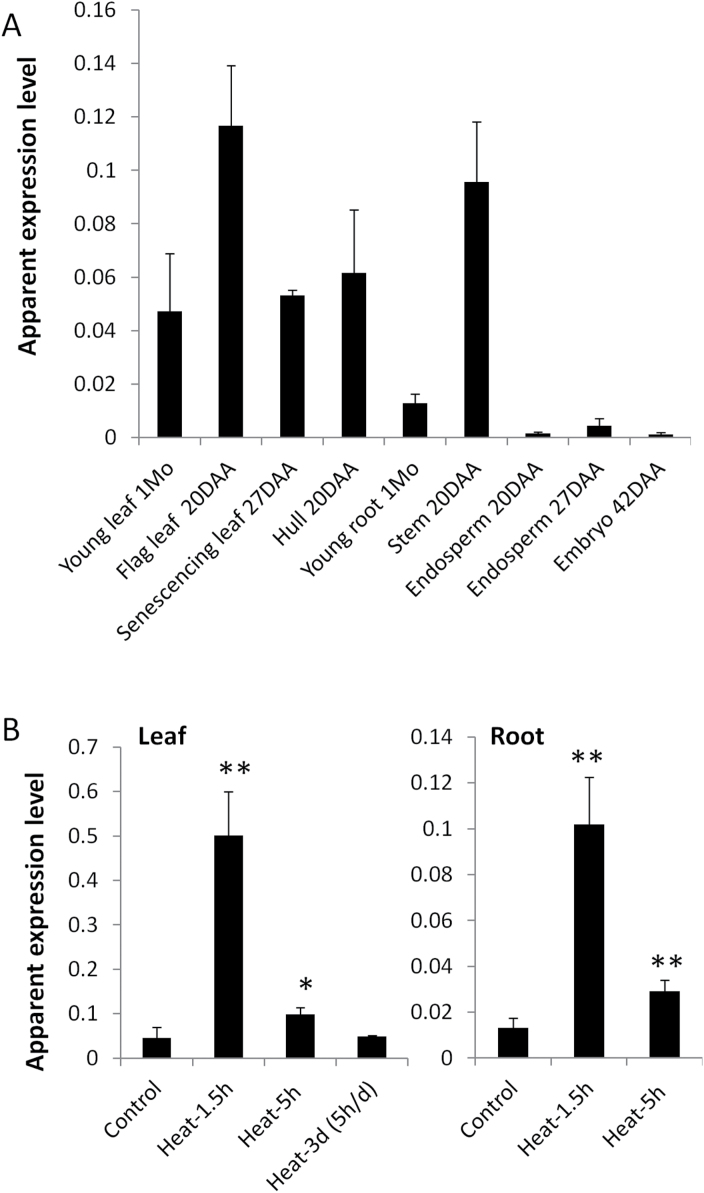
Relative mRNA abundance of *TaHsfA6f* in wheat organs and its expression response to heat stress. Values are means+SD of three biological replicates and are expressed as apparent expression levels relative to a house-keeping gene *TaRP15*. (A) Expression of *TaHsfA6f* in various organs of wheat grown under normal conditions. 1Mo, 1-month-old; 20–42DAA, 20–42 d after anthesis. (B) Expression response of *TaHsfA6f* to heat stress in one-month-old plants. Statistical significance of differences between control and heat-treated groups (36 °C for 1.5h, 5h, or 3 d with 5-h heat exposure per day) was analysed using Student’s *t*-test and is indicated by asterisks (*P<0.05; **P<0.01).

A full-length Hsf cDNA was isolated based on the EST sequence that contains the N-terminal half of the Hsf DNA-binding domain. Phylogenetic analysis based on conserved DNA-binding domain and HR region sequences showed that this cDNA encoded a Hsf protein that clustered with subclass HsfA6 members and was therefore designated as TaHsfA6f (Supplementary Fig. S2a). A full-length sequence alignment of TaHsfA6f with other TaHsfA members is shown in Supplementary Figure S2b. The *TaHsfA6f* transcript was constitutively expressed in various wheat organs with the highest expression in the mature flag leaf (Fig. 1A). Upon heat stress, the *TaHsfA6f* mRNA level was markedly up-regulated in both wheat leaves and roots (Fig. 1B). The heat up-regulation of *TaHsfA6f* was attenuated in response to prolonged heat treatment (Fig. 1B), in a similar pattern with those of other *TaHsfA* members (Xue *et al.*, 2014).

### Overexpression of *TaHsfA6f* up-regulates expression of HSP and other classes of heat stress protection genes including Golgi anti-apoptotic protein, Rof1, and Rubisco activase

**Fig. 2.**
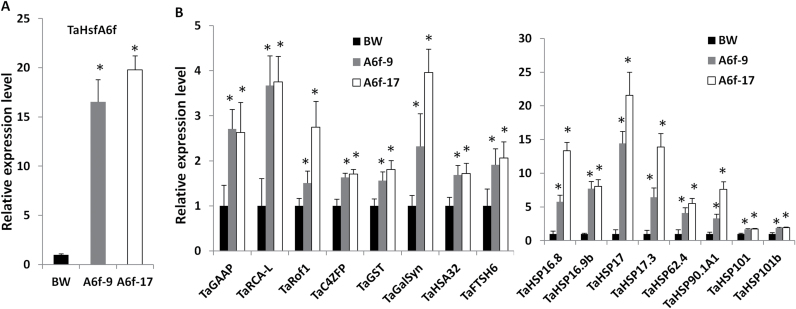
Up-regulated genes in transgenic lines overexpressing *TaHsfA6f* (A6f-9 and A6f-17). The *TaHsfA6f* transgene was under the control of the drought-inducible *HVA1s* promoter. Wheat seedlings were treated with 15% of PEG for 3 d to induce the expression of the *TaHsfA6f* transgene. Relative expression levels of genes were determined in wheat seedlings using quantitative RT-PCR. *TaHsfA6f* expression was measured as the total transcript level of both endogenous and transgene *TaHsfA6f* using primers corresponding to its coding region. TaHsfA6f up-regulated genes were selected based on our Affymetrix array data presented in Supplementary Table S2. Values are means+SD of three biological replicates. Statistical significance (*P*<0.05) of differences between Bobwhite (BW) control and transgenic lines is indicated by an asterisk. (A) Total transcript level of *TaHsfA6f*. (B) Transcript levels of genes up-regulated by *TaHsfA6f*.

To elucidate its regulatory role in modulating the expression of genes involved in wheat adaptation to heat stress, transgenic lines overexpressing *TaHsfA6f* driven by a barley *HVA1s* promoter were investigated. The barley *HVA1s* promoter is drought-inducible (Xiao and Xue, 2001). A drought-inducible promoter, instead of a heat-inducible one, was used to assist in the identification of TaHsfA6f target genes in the transgenic lines, as most heat-inducible downstream genes are not, or are little, affected by drought stress based on our analysis of publicly available Affymetrix wheat array dataset (accession # TA23 at http://www.plexdb.org, Aprile *et al.*, 2009). A total of eight transgenic lines carrying *HVA1s* promoter-driven *TaHsfA6f* were generated. Preliminary quantitative RT-PCR analysis in the seedlings of T1 transgenic lines showed that four transgenic lines had *TaHsfA6f* expression level >5-fold higher than wild-type Bobwhite plants under polyethylene glycol (PEG)-induced dehydration conditions. In addition, a heat-inducible HSP gene*, TaHSP17* (Xue *et al.*, 2014), in these four T1 transgenic lines with PEG induction of the *TaHsfA6f* transgene was 2.5–16 times higher than Bobwhite, indicating that TaHsfA6f is a positive regulator of HSP genes. At the T2 stage, two *TaHsfA6f* lines (A6f-9 and A6f-17) showed >15 times higher level of *TaHsfA6f* expression than Bobwhite under PEG-induced dehydration conditions (Fig. 2A). These two lines together with the wild-type Bobwhite were then used for identification of potential TaHsfA6f target genes using Affymetrix Wheat Genome Array.

This study focused on TaHsfA6f up-regulated genes. This is based on that *TaHsfA6f* overexpression up-regulated *TaHSP17* expression and that an initial transactivation analysis using a *TaHSP90.1-A1* promoter-driven reporter gene showed TaHsfA6f acting as a transcriptional activator (also see the section below). Affymetrix array expression profiling revealed that a total of 50 Affymetrix probesets were significantly up-regulated in the *TaHsfA6f* lines (A6f-9 and A6f-17), at least 1.5-fold higher than Bobwhite (Supplementary Table S2). These up-regulated probesets are also heat-inducible based on the analysis of a publicly available Affymetrix dataset reported by Qin *et al.* (2008). Of these up-regulated probesets, 22 were HSP genes. In addition, the mRNA levels of two more HSP genes (*TaHSP17.3* and *TaHSP90.1-A1*) were >10 times higher in transgenic lines than in Bobwhite, although the differences between transgenic plants and Bobwhite in the Affymetrix dataset were not statistically significant. These two HSP genes are also listed in Supplementary Table S2. *GAAP*, *Rof1*, *HSA32*, and *Rubisco activase large isoform* (*RCA-L*) as well as other stress protection genes [e.g. *galactinol synthase* (*GalSyn*), *glutathione-S-transferase* (*GST*), and *desiccation-related PCC13-62 protein*] were also up-regulated in the transgenic lines. Other up-regulated genes include a C4 zinc finger transcription factor (*C4ZFP*), calmodulin-like protein, and peptidase genes (*peptidase M50* and *FTSH6*). The rest of the genes up-regulated by TaHsfA6f are shown in Supplementary Table S2.

To confirm the results from the Affymetrix array analysis, 15 representative up-regulated genes, and another TaHSP gene (*TaHSP16.9b*) that has no probeset in the Affymetrix wheat genome array were selected for further expression analysis using quantitative RT-PCR. All of these genes showed a significant increase in expression levels in the high *TaHsfA6f*-expressing transgenic lines (A6f-9 and A6f-17) under PEG-induced dehydration conditions (Fig. 2B). The expression levels of *TaGAAP*, *TaRCA-L*, *TaRof1*, and *TaGalSyn* were more than 2.5 times higher in the highest *TaHsfA6f*-expressing line (A6f-17) than Bobwhite. More than 10-fold up-regulation of *TaHSP16.8*, *TaHSP17*, and *TaHSP17.3* expression levels was observed in the A6f-17 line (Fig. 2B).

**Fig. 3.**
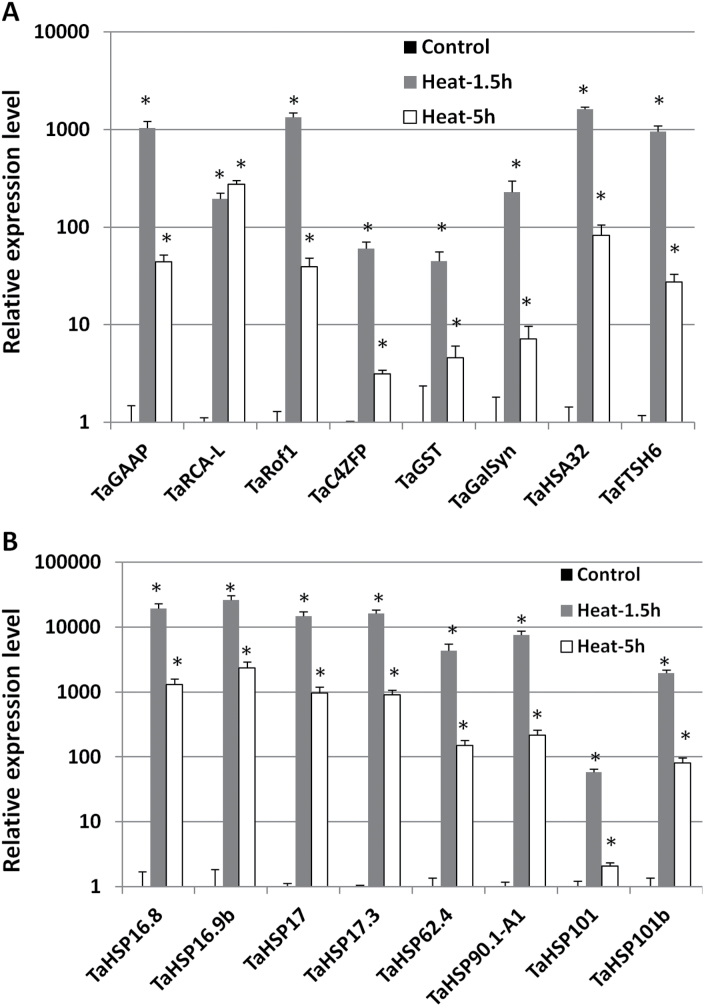
Heat-responsive expression patterns of genes up-regulated by TaHsfA6f (A and B). Expression responses were examined in the leaves of 1-month-old Bobwhite plants. Values are means+SD of three biological replicates. Statistical significance (*P*<0.05) of differences between control and heat-treated groups (36 °C for 1.5h or 5h) is indicated by an asterisk.

To investigate the heat-responsive patterns of these TaHsfA6f up-regulated genes, we determined their relative expression levels in the leaves of 1-month-old Bobwhite plants treated at 36 °C for 1.5h or 5h in comparison with plants without heat treatment. Many of these genes (*TaGAAP*, *TaRof1*, *TaHSA32*, *TaFTSH6*, *TaHSP16.8*, *TaHSP16.9b*, *TaHSP17*, *TaHSP17.3*, *TaHSP62.4*, *TaHSP90.1-A1*, and *TaHSP101b*) showed a 1000-fold or more increase in expression levels after 1.5-h heat treatment (Fig. 3). About 200-fold or more increase in expression levels was observed for *TaRCA-L* and *TaGalSyn*, and a more than 45-fold increase was observed for *TaGST*, *TaC4ZFP*, and *TaHSP101*. The heat-responsive patterns of all these genes except *TaRCA-L* during the period of 5-h heat treatment were similar to that of *TaHsfA6f* (Fig. 1B); i.e. a rapid increase at 1.5h followed by a marked attenuation at 5h (Fig. 3). *TaRCA-L* expression remained high after 5-h heat treatment (Fig. 3A).

The effect of PEG treatment on the expression of *TaHsfA6f* and its target genes was also examined in wild-type plants. PEG treatment for 3 d did not significantly affect the expression of *TaHsfA6f*, *TaC4ZFP*, *TaGST*, and *TaHSP17* in Bobwhite plants, but resulted in around 2-fold up-regulation of many genes targeted by TaHsfA6f (Supplementary Figure S3). *TaFTSH6* was the only gene that showed a marked increase in mRNA level by PEG-induced dehydration and *TaRCA-L* was down-regulated by PEG. The expression profiles of *TaHsfA6f* and its target genes indicate that these genes except *TaFTSH6* are mainly involved in heat adaptation.

### TaHsfA6f is a transcriptional activator involved in regulation of heat-induced expression of *TaGAAP*, *TaRof1*, and *TaHSP* genes

**Fig. 4.**
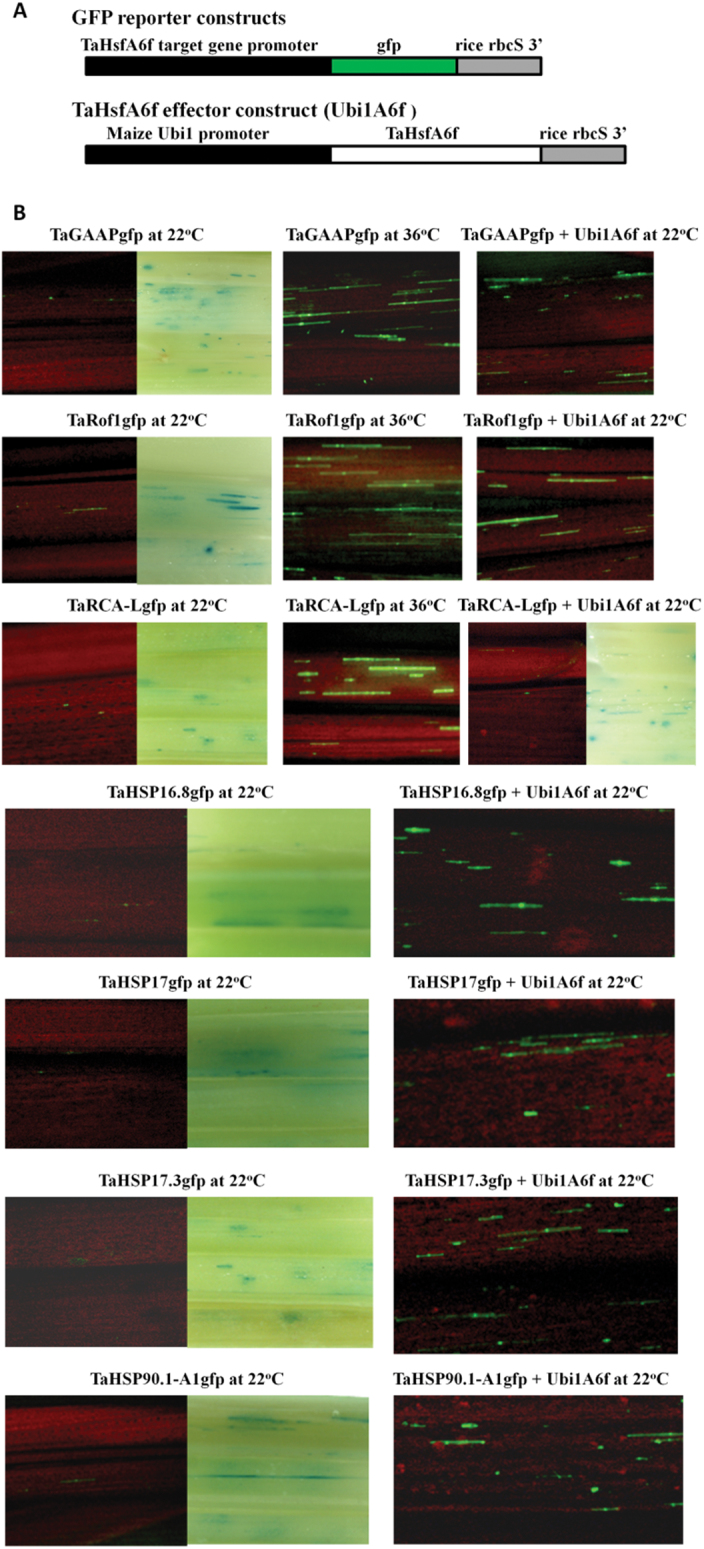
Transactivation analysis of *GFP* reporter genes driven by the promoters of TaHsfA6f target genes in wheat seedlings. (A) Reporter and effector constructs. (B) Transactivation analysis. GFP foci (green) indicate *GFP* reporter gene expression. Blue foci resulted from the expression of a co-introduced Ubi1:GUS+ reporter and indicate that tissue sections were transformed with these constructs. The red background is the fluorescence from shoot chlorophyll. The *GFP* expression of all these reporter constructs was induced at 36 °C, including those driven by *TaHSP* promoters (Supplementary Fig. S2). The *TaHsfA6f* effector construct activated the expression of reporter genes driven by *TaGAAP, TaRof1*, and *TaHSP* promoters, but not *TaRCA-L* promoter at 22 °C. Illustration of GUS foci is given only when *GFP* reporter expression is essentially undetectable in the tissue section.

To identify target genes directly regulated by TaHsfA6f, the promoter sequences of seven genes up-regulated by TaHsfA6f were successfully identified through sequence assembly from a partial wheat genome sequence database in CerealDB (Wilkinson *et al.*, 2012) and were subsequently isolated. The isolated promoters were used to drive the expression of the green fluorescence protein (*GFP*) reporter gene (Fig. 4A). The *TaHsfA6f* effector construct was driven by a constitutive maize *Ubi1* promoter. Transactivation analysis was performed by bombarding reporter and effector constructs into wheat seedlings. As shown in Fig. 4B, the expression of the *GFP* reporter gene driven by the promoter of *TaGAAP* or *TaRof1* was induced by heat stress or by co-introduction with the *TaHsfA6f* effector construct without a heat treatment. In contrast, *TaRCA-L* promoter-driven reporter was inducible by heat, but not by TaHsfA6f. The *GFP* reporter driven by a HSP promoter (*TaHSP16.8*, *TaHSP17*, *TaHSP17.3*, or *TaHSP90.1-A1*) was also strongly induced by heat treatment at 36 °C (Supplementary Fig. S4) or by TaHsfA6f (Fig. 4B). These data indicate that all of the analysed genes except *TaRCA-L* are potentially direct targets of TaHsfA6f.

To examine whether transactivation of these target genes is unique to TaHsfA6f, three TaHsfA6f target genes (*TaGAAP*, *TaRof1*, and *TaHSP17.3*) were chosen for analysis of TaHsfA1b and TaHsfA4e transactivation activity in comparison with TaHsfA6f. As shown in Supplementary Figure S5, all three effector genes were able to transactivate these three reporter genes. However, the transactivation strength of TaHsfA1b and TaHsfA4e for *TaGAAP* and *TaRof1* reporter genes was visibly weaker than that of TaHsfA6f, as assessed by relative intensity of GFP foci. In contrast, no visible differences in the intensity of GFP foci of *TaHSP17.3* reporter were observed between TaHsfA6f and TaHsfA1b or TaHsfA4e.

### TaHsfA6f binds to the elements present in the promoters of *TaGAAP*, *TaRof1*, and *TaHSP* genes

**Fig. 5.**
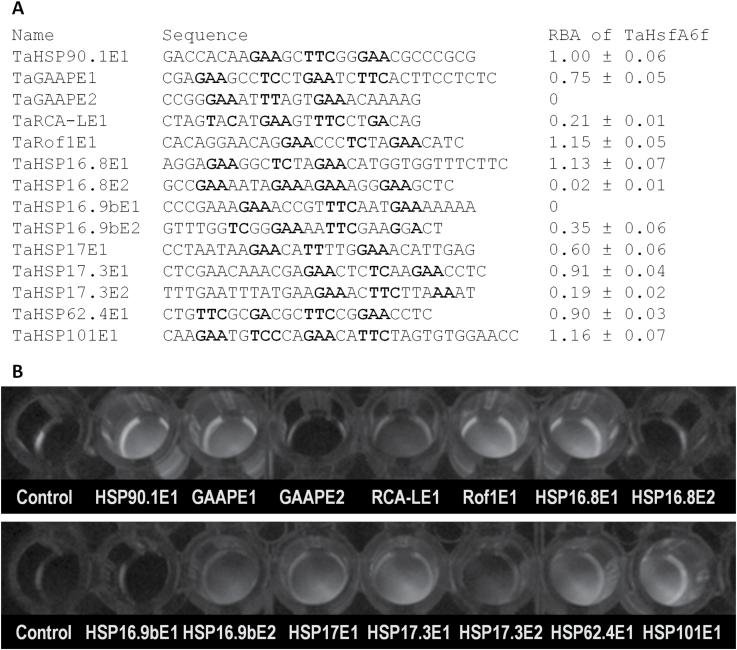
TaHsfA6f binding to HSE-like sequences in the promoters of genes regulated by TaHsfA6f. (A) Relative binding activity (RBA) of TaHsfA6f–CELD fusion protein to HSE-like sequences using CELD reporter-based DNA-binding assays. Values are means±SD of three assays and are relative to the binding activity of TaHSP90.1E1 from the *TaHSP90.1-A1* promoter, which is arbitrarily set as 1. Putative HSE sequences were selected from the available promoter sequences within 1.5kb upstream of the translation start of these genes. GAA or TTC sequences are in bold. The positions of these HSE and HSE-like elements in their respective promoters are indicated in Supplementary Fig. S1. (B) Image of a representative set of assays for TaHsfA6f binding activity. Binding activity was quantified by measuring the fluorescence of the 4-methylumbelliferyl group produced through hydrolysis of 4-methylumbelliferyl-β-d-cellobioside by TaHsfA6f–CELD, which was bound to HSE-containing oligonucleotides immobilized in wells. The control is an oligonucleotide without a HSE.

To provide further evidence that *TaGAAP*, *TaRof1*, and *TaHSP* genes are direct target genes of TaHsfA6f, the promoter sequences of 10 genes [above seven genes used in transactivation analysis plus three additional HSP genes (*TaHSP16.9b*, *TaHSP62.4*, and *TaHSP101*)] were analysed to identify whether they contain HSE elements. HSE-like motifs were identified in the promoters of all 10 genes (Fig. 5). TaHsfA6f-binding HSEs present in these promoters were subsequently identified by *in vitro* DNA-binding assays using a CELD reporter system (Xue, 2002). Although the typical HSE (GAAnnTTCnnGAA or TTCnnGAAnnTCC) motif was found only in the promoter of *TaHSP90.1-A1*, many HSE-like elements (TaGAAPE1, TaRof1E1, TaHSP16.8E1, TaHSP17.3E1, TaHSP62.4E1, and TaHSP101E1) present in these promoters had a high binding affinity for TaHsfA6f (Fig. 5). However, the TaHsfA6f binding affinity to the HSE-like element from the *TaRCA-L* promoter (TaRCA-LE1) was low. A close examination of high-affinity TaHsfA6f motifs in Fig. 5A revealed that TaHsfA6f effectively bound to a sequence of GAAnnCTCnnGAA (e.g. TaRof1E1 and TaHSP16.8E1). These DNA-binding data together with transactivation and expression data suggest that *TaGAAP*, *TaRof1*, *TaHSP16.8*, *TaHSP17*, *TaHSP17.3*, and *TaHSP90.1-A1* are direct targets of TaHsfA6f, whereas *TaRCA-L* is likely to be an indirect target.

### Transactivation of *TaHSP90.1-A1* and *TaGAAP* reporter genes by TaHsfA6f relies on the presence of a TaHsfA6f-binding element in their promoters

**Fig. 6.**
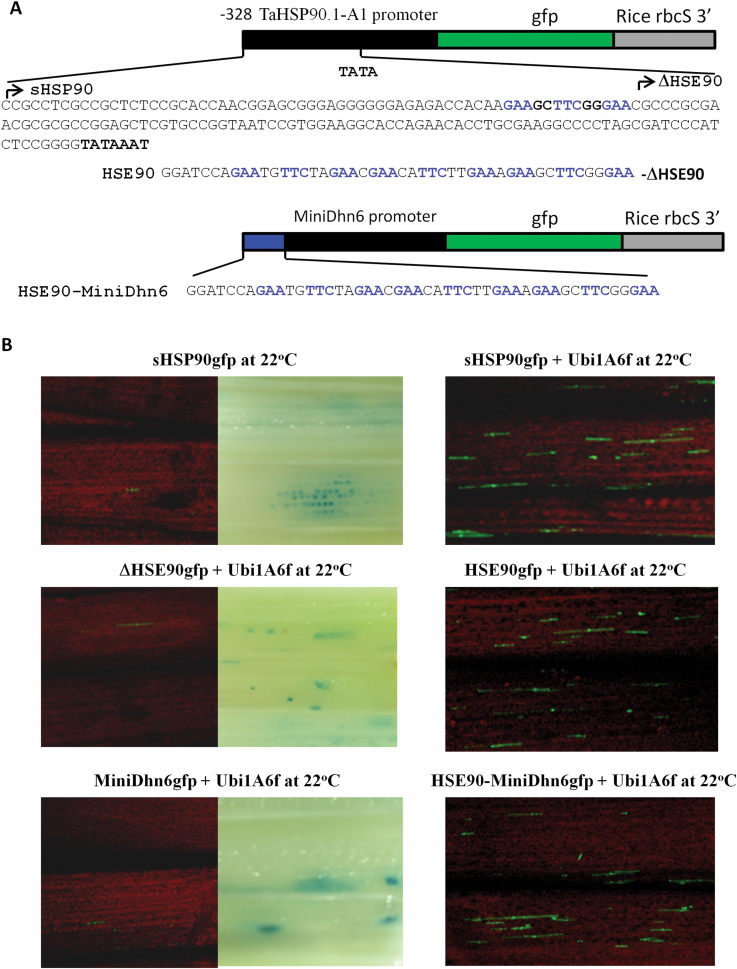
Functional analysis of the *TaHSP90.1-A1* promoter and TaHSP90.1E1 HSE (**GAA**GC**TTC**GG**GAA**) in wheat seedlings. (A) Reporter gene constructs. A short *TaHSP90.1-A1* promoter (sHSP90) has a 328-bp fragment upstream of the translational start codon, which contains TaHSP90.1E1. The sequence upstream of the TATA box of *TaHSP90.1-A1* promoter is shown. The ΔHSE90 promoter starts immediately downstream of the TaHSP90.1E1 HSE. HSE90 construct contains three TaHSP90.1E1 HSE repeats, which are added immediately upstream of the ΔHSE90 promoter. The HSE90-miniDhn6 construct was made by adding three TaHSP90.1E1 HSE repeats to a minimal promoter of the drought-inducible *Dhn6* gene. (B) Transactivation analysis. A *GFP* reporter gene was introduced into the shoots of wheat seedlings with or without the *TaHsfA6f* effect construct (Ubi1A6f). A Ubi1:GUS+ reporter gene was also co-introduced. Illustration of GUS foci is given only when *GFP* reporter expression is essentially undetectable in the tissue section.

To assess whether transactivation of the reporter genes by TaHsfA6f occurs through its binding to HSE present in the promoters of the reporter genes, promoter truncation and mutation analyses of *TaHSP90.1-A1* and *TaGAAP* were performed. A truncated *TaHSP90.1-A1* promoter (a 328-bp fragment, named as sHSP90) containing a TaHSP90.1E1 HSE was still functional for TaHsfA6f-mediated transactivation of the reporter gene (Fig. 6). A further deletion of a 64-bp fragment with the removal of TaHSP90.1E1 (ΔHSE90) abolished the TaHsfA6f-mediated transactivation of the *GFP* reporter gene (Fig. 6B) or the heat-inducible promoter activity as shown previously (Xue *et al.*, 2014). Addition of the TaHSP90.1E1 element to the ΔHSE90 promoter-driven *GFP* reporter gene restored transactivation by TaHsfA6f. TaHsfA6f was also able to transactivate a reporter gene driven by a minimal promoter (MiniDhn6) from a barley drought-inducible promoter (*Dhn6*) with the addition of TaHSP90.1E1 (HSE90-MiniDhn6) (Fig. 6 **).**

**Fig. 7.**
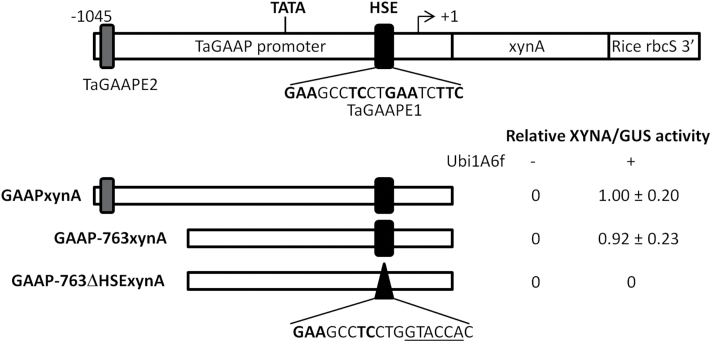
Functional analysis of the *TaGAAP* promoter in wheat seedlings by promoter truncation and site-directed mutagenesis of TaGAAPE1. A xylanase (XYNA) was used as a reporter for quantitative measurement of expression levels of reporter constructs driven by *TaGAAP* and its mutant promoters with or without the *TaHsfA6f* effector construct (Ubi1A6f). Values are means±SD of 3–4 biological replicates. Ubi1:GUS+ was used as a control gene for normalisation of transformation efficiency. Relative expression levels of the reporter gene are expressed as the ratio of XYNA to GUS activity relative to that of GAAPxynA reporter construct with the *TaHsfA6f* effector construct, which is arbitrarily set as 1. GAAP–763xynA contains a *TaGAAP* promoter fragment of 763bp upstream of the translation initiation codon. GAAP–763ΔHSExynA contains a mutated TaGAAPE1.

The promoter truncation and HSE mutation were also performed for the *TaGAAP* promoter using a xylanase (*xynA*) gene as a reporter for quantitative analysis of TaHsfA6f transactivation activity (Fig. 7). TaGAAPE1 is located downstream of a putative TATA box in the *TaGAAP* promoter. Removal of a 282-bp upstream fragment (containing TaGAAPE2) in the 1045-bp *TaGAAP* promoter (GAAP–763xynA construct) did not significantly affect its promoter activity induced by the *Ubi1* promoter-driven *TaHsfA6f* effector construct (Fig. 7). Mutation of the TaGAAPE1 element (**GAA**GC**CTC**CT**GAA**TC**TTC**) by site-direct mutagenesis to **GAA**GC**CTC**CTGGTACCAC resulted in a GAAP–763ΔHSExynA reporter construct that was no longer transactivated by TaHsfA6f (Fig. 7). These data indicate that the TaGAAPE1 element is a functional HSE required for *TaGAAP* transactivation by TaHsfA6f.

### Overexpression of *TaHsfA6f* confers thermotolerance

**Fig. 8.**
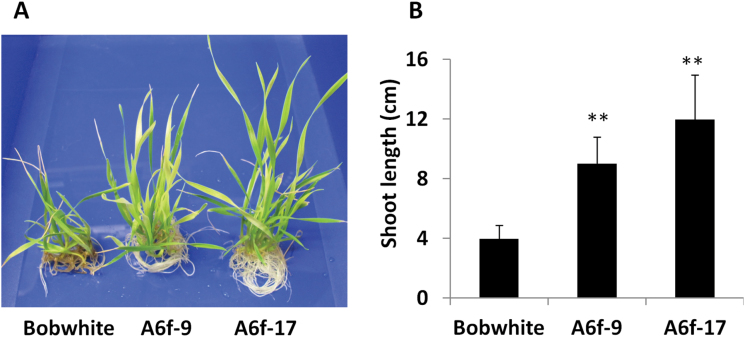
Transgenic wheat overexpressing *TaHsfA6f* showed improved thermotolerance. (A) Illustration of thermotolerance. Five-day-old seedlings of *TaHsfA6f* transgenic lines (A6f-9 and A6f-17) and Bobwhite were treated with a nutrient solution containing 15% PEG for 2 d, followed by heat shock at 45 °C for 2h, and then recovery in a controlled environment growth room for 3 weeks (15% PEG was included in a nutrient solution for the first two days of recovery). Ten seedlings of each genotype are shown. (B) Shoot length of *TaHsfA6f* transgenic lines and Bobwhite after three weeks of recovery as described above. Values are means±SD of 25–29 seedlings. Statistical significance of differences between Bobwhite control and transgenic lines is indicted by asterisks (**P<0.01).

Although the main aim of this study was to elucidate the role of TaHsfA6f in regulation of genes involved in wheat heat adaptation, relative thermotolerance of transgenic lines overexpressing *TaHsfA6f* under the control of the drought-inducible *HVA1s* promoter was also investigated using PEG-induced dehydration stress to induce the transgene expression. Both transgenic lines and wild-type Bobwhite at the 5-day-old seedling stage were pre-treated with PEG for 2 d and then exposed to heat stress at 45 °C for 2h and followed by recovery at 16 °C/20 °C (night/day) for three weeks with inclusion of further 2 d of PEG treatment at the start of a recovery period. As shown in Fig. 8, transgenic lines recovered from heat injury better than Bobwhite and had longer shoots and more newly grown roots. In contrast, without PEG treatment all seedlings from both *TaHsfA6f* transgenic lines and Bobwhite were killed by a 2-h 45 °C treatment (Supplementary Fig. S6). No difference in thermotolerance was observed between *TaHsfA6f* transgenic lines and Bobwhite when they were treated at 42 °C for 2h without PEG treatment. Furthermore, *TaHsfA6f* transgenic seedlings had similar growth to Bobwhite without any treatment or with PEG treatment only (Supplementary Fig. S6). These data indicate that the improved thermotolerance in *TaHsfA6f* transgenic lines in comparison with wild-type plants under heat- and PEG-treated conditions is attributed to induction of *TaHsfA6f* transgene expression by PEG.

It is interesting to see the improved thermotolerance in *TaHsfA6f* transgenic lines with relatively low-level mRNA accumulation of TaHsfA6f target genes (Fig. 2) in comparison with their highly induced levels at the early stage of heat induction by treatment at a heat acclimation temperature of 36 °C (Fig. 3). This may be due to the discrepancy in speed and amount between mRNA and protein accumulation in the cells during heat stress. Therefore, an experiment was performed to look at the protein level of heat induction of *TaHSP17* promoter-driven *GFP* reporter gene during the course of heat treatment. As shown in Supplementary Figure S7, no GFP foci were detected in samples treated at 45 °C or 42 °C for 2h at any monitored time points. When samples were placed at a heat acclimation temperature (36 °C), GFP foci became detectable only after 4h of the heat treatment although the mRNA levels of many HSP genes were increased by 10 000-fold after 1.5h at 36 °C (Fig. 3). We also introduced *TaHSP17* promoter-driven *GFP* reporter gene to the seedlings of the A6f-17 line that were pre-treated with 15% PEG for 2 d to induce the accumulation of TaHsfA6f transgene protein; GFP foci were also detectable after 4h of post bombardment without heat treatment. The level of GFP protein, as estimated by GFP foci intensity, was much higher in the samples that were co-bombarded with *HVA1s* promoter-driven *TaHsfA6f* effector gene with 15% PEG treatment during the 24-h post-bombardment period, compared with A6f-17 samples. This is due to that the copy number of TaHsfA6f effector gene introduced by particle bombardment in a transient expression system is much higher than that in stably transformed transgenic lines. The data from this experiment suggest that the thermotolerance level of wheat plants at above 42 °C without heat acclimation is probably based mainly on the basic levels of thermo-protectants present in the cells, as the high temperature above 42 °C apparently impaired protein synthesis in wheat cells. Pre-treatment of the *TaHsfA6f* overexpressing lines with PEG for 2 d is expected to provide a sufficient time to accumulate considerably higher TaHsfA6f target gene proteins in the cells of TaHsfA6f transgenic plants than Bobwhite during heat treatment at 45 °C, which resulted in better thermotolerance at 45 °C in the *TaHsfA6f* transgenic lines than wild-type plants.

## Discussion

This study investigated the regulatory network of TaHsfA6f in wheat. A number of HsfA subclasses are known to have a role in regulation of genes involved in heat stress protection (Mishra *et al.*, 2002; Liu and Charng, 2012; Charng *et al.*, 2007; Schramm *et al.*, 2008; Pérez-Salamó *et al.*, 2014; Kotak *et al.*, 2007*b*; Xue *et al.*, 2014). Constitutive overexpression of HsfA members from several other plant species lead to up-regulation of heat inducible genes in transgenic plants under non-stress conditions (Prändl *et al.*, 1998; Li *et al*, 2005; Nishizawa *et al.*, 2006; Ogawa *et al.*, 2007; Yokotani *et al.*, 2008; Liu and Charng, 2013). However, involvement of subclass HsfA6 members in regulation of plant adaptation to heat stress is currently unknown. In *Arabidopsis*, *HsfA6a* expression is not significantly influenced by heat, but is up-regulated by abscisic acid, salt, or drought stress (Hwang *et al.*, 2014). This study showed that TaHsfA6f was a transcriptional activator, acted as a positive regulator of several classes of heat stress protection genes, and contributed to thermotolerance in wheat. *TaHsfA6f* was expressed mainly in green organs (leaf, stem, and hull) under non-stress conditions and was highly heat-inducible during the early hours of heat stress, but not by PEG-induced dehydration stress, indicating its potential role in protecting these organs during heat stress. The heat induction pattern of *TaHsfA6f* was similar to those of the HSP genes, the expression levels of which are attenuated after prolonged heat exposure (5-h heat treatment) (Xue *et al.*, 2014). Expression analysis of transgenic lines overexpressing *TaHsfA6f* under the control of the drought-inducible *HVA1s* promoter revealed that TaHsfA6f positively regulated expression of a number of HSP and other classes of known-function heat stress protection genes that include *TaGAAP*, *TaRof1*, *TaRCA-L, TaGST*, *HSA32*, and *TaGalSyn*. Expression of all of these TaHsfA6f-up-regulated genes was strongly induced by heat treatment with a similar heat-responsive pattern to that of *TaHsfA6f* with the exception of *TaRCA-L*.

Among these TaHsfA6f-regulated genes, the role of HSPs in heat protection is well known (Wang *et al.*, 2004; Basha *et al.*, 2010). Other proteins that have been shown to have a positive impact on thermotolerance are Rof1, galactinol synthase, GST, HSA32, and RCA. Rof1 (FKBP62) is a member of the multidomain FK506 binding protein family and is known to physically interact with HSP90.1 in *Arabidopsis* (Meiri and Breiman, 2009). Overexpression of *Rof1* in *Arabidopsis* leads to improved long-term acquired thermotolerance, whereas knockout mutation of *Rof1* in *Arabidopsis* reduces thermotolerance (Meiri and Breiman, 2009). Galactinol synthase catalyses the first step in the synthetic pathways of raffinose family oligosaccharides, which are compatible solutes with a role in protecting plant cells against several abiotic stresses including heat stress (Panikulangara *et al.*, 2004). GST has a role in alleviating oxidative stress (Gill and Tuteja, 2010). Reactive oxygen species are elevated during heat stress (Mittler *et al.*, 2012), which leads to oxidative stress. HSA32 is another important protein up-regulated by heat in plants (Charng *et al.*, 2006). A knockout mutation study has shown that HSA32 is essential for long-term acquired thermotolerance in *Arabidopsis* (Charng *et al*., 2006, 2007). HSA32 can slow the turnover of HSP101 and is involved in prolonging the memory of heat acclimation (Wu *et al.*, 2013). Rubisco activase is a member of an AAA family of proteins (a class of chaperone-like ATPases) and has been considered as a specific catalytic chaperone for maintaining Rubisco in active form (Portis, 2003). High temperature promotes inactivation of Rubisco activase, which subsequently reduces the amount of the active form of the Rubisco enzyme and hence the photosynthesis rate. It has been shown that a decrease in Rubisco activase leads to increased thermosensitivity of photosynthesis (Sharkey *et al.*, 2001). Rubisco activase mRNA level has been reported to be down-regulated in cotton during heat stress (DeRidder and Salvucci, 2007), but in rice the Rubisco activase large isoform protein level is up-regulated by heat with a slight decrease in the Rubisco activase small isoform protein level (Wang *et al.*, 2010). Rubisco activase large and small isoforms in rice are derived from the same gene by alternative splicing and there is no significant change in the total Rubisco activase mRNA level in rice during heat stress (Wang *et al.*, 2010). Transgenic rice plants overexpressing a Rubisco activase large isoform showed higher thermotolerance than wild-type plants (Wang *et al.*, 2010). The NCBI sequence database search and analysis revealed two Rubisco activase genes in wheat: a large isoform (*TaRCA-L*, AF251264) and a small isoform (*TaRCA-S*, DQ984669) encoded by two separate nuclear genes. This study showed that expression of *TaRCA-L* was markedly up-regulated by heat in wheat leaves. Heat up-regulation of *TaRCA-L* expression could partly compensate its heat-promoted inactivation. In addition, during heat stress RCA has been proposed to have another chaperone role in potential protection of protein synthesis machinery in the chloroplast against heat inactivation (Rokka *et al.*, 2001).

A number of heat protection genes directly regulated by HsfAs have been experimentally demonstrated in previous studies in several plant species, which are mainly HSP genes (Rojas *et al.*, 2002; Kotak *et al.*, 2007*b*; Singh *et al.*, 2012; Xue *et al.*, 2014) and galactinol synthase (Panikulangara *et al.*, 2004; Pillet *et al.*, 2012). This study identified a TaHsfA6f regulatory network and a number of direct target genes regulated by TaHsfA6f. Analysis of promoter sequences of ten TaHsfA6f up-regulated genes revealed that all these genes, except *TaRCA-L* and *TaHSP16.9b*, contain at least one high-affinity TaHsfA6f-binding element. TaHsfA6f showed a high binding affinity to the typical HSE (GAAnnTTCnnGAA) as well as a sequence of GAAnnCTCnnGAA. Results obtained from transactivation analysis of reporter genes driven by the promoters of *TaHSP16.8*, *TaHSP17*, *TaHSP17.3*, *TaHSP90.1-A1*, *TaGAAP*, and *TaRof1* also support that these TaHsfA6f up-regulated genes are direct target genes of TaHsfA6f. The reporter gene driven by the *TaRCA-L* promoter was also heat-inducible, but not by TaHsfA6f in transactivation analysis. The transactivation data together with the absence of a high-affinity TaHsfA6f element in the *TaRCA-L* promoter suggest that *TaRCA-L* is likely to be an indirect target gene of TaHsfA6f.

Most significantly, two direct TaHsfA6f target genes (*TaRof1* and *TaGAAP*) identified in this study have not been experimentally demonstrated previously for any Hsf members from other plant species. Comparative analysis of relative transactivation strength of TaHsfA6f with TaHsfA1b and TaHsfA4e showed that *TaGAAP* and *TaRof1* reporter genes were preferentially regulated by TaHsfA6f among these three TaHsfA subclass members. TaRof1 protein shares 73.3% and 91.7% amino acid identities with previously characterised wFKBP73 and wFKBP77 in wheat (Blecher *et al.*, 1996; Kurek *et al.*, 1999). wFKBP77 is also a heat-inducible gene, whereas wFKBP73 is not (Kurek *et al.*, 1999). The involvement of GAAP in plant adaptation to high temperatures has not been reported to date. Studies from mammalian systems suggest that GAAP has a role in suppressing programmed cell death (Gubser *et al.*, 2007; Saraiva *et al.*, 2013). This study showed that the *GAAP* gene in wheat was highly heat-inducible (>1000-fold increase with 1.5-h heat treatment at 36 °C) and its heat induction pattern is similar to that of *TaHSA32*. Further evidence of TaHsfA6f being a direct transcriptional activator of *TaGAAP* and *TaHSP90.1-A1* was from promoter truncation and HSE mutation analyses. Transactivation of *TaGAAP* and *TaHSP90.1-A1* by TaHsfA6f relied on the presence of a high-affinity TaHsfA6f-binding HSE in their promoters. These analyses provide substantial evidence that TaHsfA6f is a transcriptional activator that directly regulates the heat-inducible expression of *TaGAAP* and *TaHSP90.1-A1*. Furthermore, this study showed that the addition of the TaHSP90.1E1 element to a drought-inducible promoter resulted in a reporter gene that was inducible by heat or TaHsfA6f, thus demonstrating that a functional HSE is sufficient for turning a non-heat-inducible promoter into a heat-inducible one.

Another finding of this study is the identification of a gene that encodes a highly heat-inducible transcription factor (TaC4ZFP) from the ZPR1 zinc finger family and which is regulated by TaHsfA6f. *TaC4ZFP* transcript level was up-regulated by 60-fold after 1.5-h heat treatment in wheat leaves. TaC4ZFP contains two C4-type zinc fingers, known as the ZPR1 domain. Recently, a ZPR1 protein from wild tomato has been shown to be a transcriptional activator and binds to an ABA-responsive element (Li *et al.*, 2013). Regulation of this family of transcription factors by a Hsf is previously unknown. Future work on characterisation of the TaC4ZFP regulatory network would identify further downstream genes of TaHsfA6f.

It is interesting to note that there was a large difference in the accumulation of target gene transcript levels between early heat induction of target genes in wild-type plants and *TaHsfA6f* overexpressing transgenic plants, although *TaHsfA6f* expression levels in transgenic plants (A6f-9 and A6f-17) carrying *HVA1s* promoter-driven *TaHsfA6f* transgene under PEG-induced dehydration conditions were almost comparable to its heat-induced level. This discrepancy may be explained by the following two factors. Firstly, many heat-up-regulated genes are regulated by multiple members of the Hsf family. In particular, TaHsfA2 subclass members are phylogenetically close to TaHsfA6 members. A TaHsfA2 member (TaHsfA2b) has also been shown to be a strong transcriptional activator for activation of expression of *TaHSP17* and *TaHSP90.1-A1* in wheat (Xue *et al.*, 2014). Secondly, it is known that Hsf repressors are active under non-heat stress temperature (Hahn *et al.*, 2011; Scharf *et al.*, 2012), which will reduce the level of functional TaHsfA6f protein in TaHsfA6f transgenic plants. Although the transcript levels of TaHsfA6f target genes in transgenic lines after 3 d of PEG treatment were relatively low, improved thermotolerance of transgenic lines overexpressing *HVA1s* promoter-driven *TaHsfA6f* was observed at a non-acclimation high temperature (45 °C). It is likely that synthesis of heat protection proteins is impaired at this high temperature in wheat cells, as no GFP foci of the *TaHSP17*-promoter-driven reporter gene was visible at temperatures above 42 °C. In addition, in the experiment presented in Fig. 8, we included a further 2-day PEG treatment during the post-heat recovery period, which can result in higher protein levels of TaHsfA6f target genes in TaHsfA6f transgenic lines than wild-type plants and can help the recovery of heat-injured plants.

In conclusion, this study, through the identification of TaHsfA6f target genes, shows that TaHsfA6f is one of the regulators of wheat adaptation to heat stress. This is supported by observation of improved thermotolerance of plants overexpressing *TaHsfA6f*. It is likely that the improved thermotolerance observed in the TaHsfA6f transgenic plants relies on the concerted action of genes regulated by this transcriptional activator. This subclass HsfA6 regulatory network includes three important classes of previously unknown targets: GAAP, RCA-L, and C4ZFP. For improvement of thermotolerance in crop species grown in heat-prone environments, an appropriate HsfA overexpression system needs to be investigated, as constitutive overexpression of a Hsf gene can lead to growth retardation (Ogawa *et al.*, 2007; Zhu *et al.*, 2009). This study used a drought-inducible expression system for overexpression of *TaHsfA6f*, which may be useful for wheat crops grown in terminal drought stress environments where wheat crops often encounter soil water deficit and occasional heat stress during grain filling. However, the expression level of the *TaHsfA6f* transgene using the drought-inducible promoter obtained in this study may not be high enough to achieve marked improvement of thermotolerance. Furthermore, the usefulness of this approach for improvement of wheat yield under drought/heat-prone environments awaits further investigation in field trials with appropriate field growth conditions.

## Supplementary data

Supplementary data are available at *JXB* online.

Table S1. Real-time-PCR primers of *T. aestivum* genes.

Table S2. Genes are up-regulated in TaHsfA6f over- expressing lines.

Figure S1. Analysed promoter sequences of heat up-regulated genes from *T. aestivum*.

Figure S2. Neighbor-joining phylogenetic tree of wheat HsfA proteins and amino acid sequence alignment of TaHsfA6f with other full-length TaHsfA class members.

Figure S3. Expression of *TaHsfA6f* and its target genes in wild-type plants in response to PEG treatment.

Figure S4. The heat-induced expression of *GFP* reporter genes driven by HSP promoters.

Figure S5. Relative strength of TaHsfA6f transactivation activity in comparison with TaHsfA1b and TaHsfA4e.

Figure S6. Illustration of thermotolerance and growth of *TaHsfA6f* transgenic lines (A6f-9 and A6f-17) and Bobwhite (BW) under various control conditions.

Figure S7. Time course of the appearance of GFP foci produced by *TaHSP17* promoter-driven *GFP* reporter gene under various conditions.

Supplementary Data

## Abbreviations:

C4ZFP: C_4_ zinc finger transcription factor
GAAP: Golgi anti-apoptotic protein
GalSyn: galactinol synthase
GFP: green fluorescence protein
GST: glutathione-*S*-transferase
Hsf: heat shock factor
HSP: heat shock protein
RCA-L: Rubisco activase large isoform.
