# Supplementary material for: TaHsfA6f is a transcriptional activator that regulates a suite of heat stress protection genes in wheat (Triticum aestivum L.) including previously unknown Hsf targets
Source: J Exp Bot. 2014 Nov 26;66(3):1025–39. doi: 10.1093/jxb/eru462 (PMC4321556; doi:10.1093/jxb/eru462)
Supplement: Supplementary Data [file supp_66_3_1025__index.html]

TaHsfA6f is a transcriptional activator that regulates a suite of heat stress protection genes in wheat (Triticum aestivum L.) including previously unknown Hsf targets — TaHsfA6f is a transcriptional activator that regulates a suite of heat stress protection genes in wheat (Triticum aestivum L.) including previously unknown Hsf targets — TaHsfA6f is a transcriptional activator that regulates a suite of heat stress protection genes in wheat (Triticum aestivum L.) including previously unknown Hsf targets — Supplementary Data 

# TaHsfA6f is a transcriptional activator that regulates a suite of heat stress protection genes in wheat (*Triticum aestivum* L.) including previously unknown Hsf targets

## Supplementary Data

Data files

**Files in this Data Supplement:**

- Supplementary Data - Supplementary Data
