# Supplementary material for: TaHsfA6f is a transcriptional activator that regulates a suite of heat stress protection genes in wheat (Triticum aestivum L.) including previously unknown Hsf targets
Source: J Exp Bot. 2014 Nov 26;66(3):1025–39. doi: 10.1093/jxb/eru462 (PMC4321556; doi:10.1093/jxb/eru462)
Supplement: Supplementary Data [file supp_eru462_jexbot130476_file001.pdf]

Supplementary data

TaHsfA6f is a transcriptional activator, regulating a suite of heat stress protection genes in wheat (*Triticum aesticum* L.) including previously unknown Hsf targets

Gang-ping Xue, Janneke Drenth and C. Lynne McIntyre
CSIRO Plant Industry, 306 Carmody Road, St Lucia, Qld 4067 Australia

Supplementary Table S1. Real-time-PCR primers of *T. aestivum* genes

Supplementary Table S2. Genes are upregulated in TaHsfA6f over-expressing lines.

Supplementary Figure S1. Analysed promoter sequences of heat upregulated genes from *T. aestivum*.

Supplementary Figure S2. Neighbor-joining phylogenetic tree of wheat HsfA proteins and amino acid sequence alignment of TaHsfA6f with other full-length TaHsfA class members.

Supplementary Figure S3. Expression of *TaHsfA6f* and its target genes in wild-type plants in response to PEG treatment.

Supplementary Figure S4. The heat-induced expression of *GFP* reporter genes driven by HSP promoters.

Supplementary Figure S5. Relative strength of TaHsfA6f transactivation activity in comparison with TaHsfA1b and TaHsfA4e.

Supplementary Figure S6. Illustration of thermotolerance and growth of *TaHsfA6f* transgenic lines (A6f-9 and A6f-17) and Bobwhite (BW) under various control conditions.

Supplementary Figure S7. Time course of the appearance of GFP foci produced by *TaHSP17* promoter-driven *GFP* reporter gene under various conditions.

Supplementary Table S1. Real-time-PCR primers of *T. aestivum* genes*

Gene	Forward primer	Reverse primer
TaHsfA6f	5′-ACGACTTCTGGGAGGAGCTG	5′-TCTCCTCCTTTCCAGCTCTAAAATC
TaC4ZFP	5′-GAATGAGGAGCTTGGCTTGAA	5′-CAGGCTACAACACTGAGCACAAA
TaFTSH6	5′-GAGTTCAGGGCGATCCTGTC	5′-CAAGCAGTGACCATGTCTGTCC
TaGAAP	5′-GGCATCCGATTCCTGAAGTG	5′-ACACACAAGCAAATCACATTCGT
TaGalSyn	5′-GATCCTCGTCAGGAACTGGTG	5′-GGCCAGGCCCTTGAAATC
TaGST	5′-ATTTCGCATCGTGGGATCAG	5′-CACACACGCAAAGCACAAAA
TaHSP16.8	5′-GGGTTGGAGCCAGTTTCGT	5′-CATTTATCCCTCTGTTGGCGATT
TaHSP16.9b	5′-GCCAAGGTGGAGGAGGTG	5′-CTCGCACACACATCAACCAAA
TaHSP17	5′-GTGCGAGCTCTGCCACCT	5′-GTACAGATGCCTCGCCACAC
TaHSP17.3	5′-CGTCCAGATCACGGGGTAGA	5′-TTCTCGCATCCTCCTCGTCT
TaHSP62.4	5′-AGGCCCTGGCCAAGATGT	5′-CTCACAAAGTCACAAGCAAAGCA
TaHSP90.1-A1	5′-GTCTCATTTCCTTGCAACCAGG	5′-CTAGACACGCAGCGACCAAC
TaHSP101	5′-GATGAAGATCATGCAGGACAGC	5′-CTGGTGCAGGCACTTGGAA
TaHSP101b	5′-AAGTGCCTGTCGGATGGAGT	5′-GTAACAGCTTCCGGCCACAC
TaHSA32	5′-AGCTCTGACATGAGCCTCAGC	5′-CACGGCAGTAGCCAGTAGGAA
TaRCA-L	5′-AGCTCGCCGACAAGTACCTC	5′-GCACGCATGCATCTTAAGTATAGG
TaRof1	5′-AGCAGGAAGCGCAGCCTAT	5′-AGGACAAGACAACCGAAACCA
TaRP15	5′-GCACACGTGCTTTGCAGATAAG	5′-GCCCTCAAGCTCAACCATAACT
TaRPII36	5′-ACGTATTAACCAAGAACTCATGGAGAC	5′-TCAAATACTTTTGTAGGGCTGCTCTC

*The gene specificity of primers for each gene during primer design was checked by blasting primer sequences against the sequences in the TaGI database (http://compbio.dfci.harvard.edu/cgi-bin/tgi/gimain.pl?gudb=wheat) using an Expect value setting of 10,000 to avoid matching to non-targeted genes.

Supplementary Table S2. Genes are upregulated in TaHsfA6f over-expressing lines. Relative expression levels of genes are Affymetrix array hybridisation values, which are presented as mean and SD of three biological replicates for Bobwhite control or two TaHsfA6f transgenic lines (A6f-9 and A6f-17). Fold change is the ratio of transgenic mean to Bobwhite mean. Statistical significance is indicated by *P*-values analysed using Student's *t*-test. Two probesets (TaAffx.73024.1.S1_at and Ta.9140.1.A1_at) that are not statistically significant are also listed because of their high values of fold changes. All these listed probesets are heat-upregulated in the leaves of wheat based on Affymetrix dataset (E-MEXP-1523) deposited in http://www.ebi.ac.uk/arrayexpress/experiments/E-MEXP-1523/ by Qin et al. (2008).

Affymetrix probeset ID	DESCRIPTION	Bobwhite control		TaHsfA6f transgenic lines		Fold change	*P*-value
		Mean	SD	Mean	SD		
Ta.28630.1.S1_at	HSP17	127	112	5933	1282	46.68	0.003
TaAffx.73024.1.S1_at	HSP17.3	128	45	3418	2592	26.66	*0.095*
Ta.28631.1.S1_at	HSP16.9c	1257	482	24861	6905	19.78	0.008
Ta.9140.2.S1_at	HSP90.1-B1	5	0	99	50	18.80	0.038
Ta.24801.1.S1_at	HSP16.8	287	190	4967	2366	17.29	0.034
Ta.6123.2.S1_at	HSP70 family protein	5	0	78	10	14.74	0.001
Ta.14573.1.S1_at	HSP17.9	201	36	2857	767	14.19	0.007
TaAffx.18332.1.S1_at	HSP70 family protein	23	9	297	27	12.92	0.000
Ta.6123.1.A1_s_at	HSP62.4	216	105	2592	30	12.03	0.000
Ta.9140.1.A1_at	HSP90.1-A1	32	1	358	230	11.03	*0.075*
Ta.4877.1.S1_at	HSP70 family protein	9	3	87	13	9.37	0.002
Ta.12180.1.S1_at	Peptidase M50 family protein	98	38	830	16	8.44	0.000
Ta.6123.1.A1_x_at	HSP70 family protein	4	0	35	17	8.11	0.040
Ta.9454.1.S1_at	Desiccation-related protein PCC13-62	133	51	911	121	6.83	0.002
Ta.6123.1.A1_at	HSP70 family protein	4	0	30	12	6.82	0.029
Ta.5757.1.S1_at	Glutamine amidotransferases class-II	436	222	2852	1350	6.54	0.045
TaAffx.13929.1.A1_at	Phospholipase D delta	16	4	88	10	5.43	0.001
Ta.27661.1.S1_at	HSP17.6	363	69	1819	492	5.01	0.012
TaAffx.93351.1.A1_at	HSP70 family protein	50	1	249	54	4.99	0.006
Ta.3960.1.S1_at	Golgi anti-apoptotic protein (GAAP)	70	24	343	69	4.87	0.007
TaAffx.51944.1.S1_x_at	HSP17.6	24	3	104	28	4.36	0.013
Ta.6111.1.S1_at	Galactinol synthase (GalSyn)	561	125	2217	589	3.95	0.015
TaAffx.100380.1.A1_at	ATP-dependent peptidase (FTSH6)	93	28	345	64	3.71	0.008
Ta.27661.2.S1_at	HSP17.6b	27	2	97	34	3.65	0.029
Ta.1404.1.S1_at	Ribulose bisphosphate carboxylase/oxygenase activase large isoform (RCA-L)	383	5	1240	20	3.24	0.000
TaAffx.24372.1.S1_at	HSP17.6-like	21	5	61	11	2.85	0.010
Ta.14019.1.S1_s_at	Unknown protein	70	35	200	21	2.84	0.019
Ta.256.1.S1_at	HSP101b	157	67	428	60	2.73	0.019
Ta.639.1.S1_at	FKBP62 (Rof1)	530	27	1407	186	2.66	0.003
TaAffx.12664.1.S1_at	Unknown protein	111	30	295	34	2.65	0.007
Ta.4427.1.S1_at	HSP17.5	99	22	251	3	2.54	0.003
Ta.12067.3.A1_x_at	Carboxylesterase-like protein	22	3	57	14	2.52	0.020
Ta.2822.2.S1_at	HSA32	408	136	980	143	2.40	0.020
Ta.13370.1.A1_at	Vacuolar sorting receptor	764	80	1607	490	2.10	0.050
Ta.12067.1.A1_at	Carboxylesterase-like protein	164	46	331	37	2.02	0.024
Ta.12067.3.A1_at	Carboxylesterase-like protein	20	4	41	9	2.01	0.038
TaAffx.66113.1.S1_at	Unknown	73	24	143	23	1.96	0.047
Ta.25345.1.A1_at	Unknown	23	0	44	5	1.92	0.003
Ta.132.1.S1_a_at	HSP17.5	5806	547	11050	1908	1.90	0.017
TaAffx.70031.1.A1_at	C4 zinc finger protein (C4ZFP)	315	26	587	34	1.87	0.002
Ta.7778.1.S1_at	Unknown	408	65	739	106	1.81	0.021
TaAffx.972.1.A1_at	Uncharacterized protein family, UPF0114	53	7	93	0	1.76	0.005
Ta.23663.1.S1_s_at	TaHSP16.9d	83	0	146	12	1.75	0.002
Ta.10236.2.S1_a_at	Cysteine-rich receptor-like protein kinase	254	37	442	1	1.74	0.006
Ta.9115.1.S1_at	ClpB heat shock protein-like	2436	77	4217	218	1.73	0.001
Ta.2822.1.S1_x_at	HSA32	773	179	1329	12	1.72	0.025
Ta.261.1.S1_at	HSP101	124	7	207	28	1.68	0.013
Ta.9099.1.S1_at	Calmodulin-like	306	19	512	86	1.67	0.023
TaAffx.98749.1.S1_at	Digalactosyldiacylglycerol synthase 1	156	17	258	42	1.66	0.028
Ta.24150.1.S1_at	Glutathione-S-transferase (GST)	2461	349	4020	276	1.63	0.014
Ta.7007.1.S1_at	Unknown	130	9	204	20	1.58	0.009
Ta.14095.1.S1_at	Unknown	410	59	619	75	1.51	0.039

Promoter sequences

TaGAAP (GenBank accession #: KJ685918)
ATCTGACAAATAAACTAAACCGG**GAA**AT**TTA**GT**GAA(TaGAAPE2)**ACAAAAGTTGTACACACTTTGAGTAAAACTTGCACTATCCTTCAGCATGCAAAAACTGTTGTAA
AAACCAACGGACTTGCTAAAAAATGTGTTTCAATATTTGCGTATCTTTATCATTTTGTAAAAAAAAATTATAGTATAATGGGCTAATAGAAATTTCATTGAGATACTGA
AAATAAACACCCCAAAAACCAATTGCGCATACTTTTGTCAATTACGAGAATCATGCATTTTCCACTAGATTAACATAGAAGATTGTACCCCATACGATAAGAGAAACTG
TTCTAATAGTCAAAATAAAGAGAAATTGTTTTCCATTTGTGAAAAAAAAATATTTCAATCTTATTAATAATATGTCAACGGTACATCATATACGGAACATCCTCCCCCG
CTGTCGACAAGGCCAGCGGAGGAGGTGGAGGCAAAGGTGCGCCGCCATCATGGTCACCCTAGCGGCCATGCTTTGGTTTTTTAGAGTAACTGTTTTTTAAAGAGGAATT
TTTTAGAGTAACTGTTATGAAACTGAAAAGTTTAACTTTGCAAAGCAGGCCTATAAATAGGCCGCGCGATACTTCGAAACAGAAGGCAGGTAATCGCCTGAATGAAGCA
AAATACGGATAACCCTCGTCGGTAAGCAAAGGCATCACACATATTTTGCGGCACAACTTTCTCCTCCCTCCAGACGCCACCAGAGACCAGACTTACCAGAAGGTCAGCC
ATCCCGGCAGGCCCCACGCGTCAGCCTCCCGGAAGCGCCACACGCGACCCCAACCGCCGA**GAA**GC**CTC**CT**GAA**TC**TTC(TaGAAPE1)**ACTTCCTCTCTCGCGCCATCCT
CCCTCCCGTCCTGTCCCCTGTCCTCTCCTTAAAACTCTCCGCCCCACGCCAGCTCTCACCGCACCGCCGCACAACCGCACCGGCAAAGAAACCGAGGCGACACGTGCTC
TGCTCCGGGACTCCGAACCATCCCATTCGATCCGTGCACAGCCGTAGAGGAGAGGAGAGGAAAGGAAGACCGACCACCGGCG**ATG**

TaHSP16.8 (KJ685920)
ATGATTGAGCTAAGTGGACATGTTGCTTTCTCGAAAAGTCGATATCTTACTTGTACGCTAGTCTCAAACCTTTTCATATTCGCCCAATATAATCACACGGGAAAGGTGG
AAAAAATGTACAAATGGATATAATTCATGACTATTTTCCAAAATTATGGACATAATTTAAATTATAATATTTTTTCAAAACTATGAACATTGCTTTTCAAAAAGATATC
CAGGAAGGAGTTTTCATATTAGTGAATAGTTGTTAAATTTCCAAAATTTTAAAGCCACAAACATTATTTTTTTTTTGAAAAGGAGGAAGACCCCCGGCCTCTGCATCTG
GGTGATGCATGCAGCCACAAACGCAAACATTATTTAGAATTAATGAACATTTAAAAAATCTTGATCAATTCTAAATCTCATGAACAATTTTGTAGAATTCATTGTTATT
TTTTAAAATGTGAAAACATTGTGTTAATTCGTGAATGCTTTTAAAAAAATGTGAAATTTTTCAAAAATCCACAAAAAAAATGGATTCTTGACTGCTTTAATTAAAATGT
TTAAATAGAATGTTTGTTCCGACAATGTTATGCGGGACCCATGACGTGATGTGACCTTTACATGCAGGGTCCATTGTAAGTGAAAAAAAGTTCAGAGCCCTGCTAGGCC
TCTATTCCTTGGGAGCTTCAAGAAATGACGGCAGCATGCACACAACAACTGCAGCAAACGAACAGAGCTTATTTCTGTCGTCCTCTCTCACTGTGTGTGAACTGTCGAA
CGGTGGATATGTGCACTGACAAGTGACTGTGAGTGTTTTATTAGGATTGTTGATTTGGTCTTTGATTGGTGCTCTTCTCGGTTGGTCTCAGCTCCCGTGCGAGCCGTAT
GCTCGGTGCATTCCTGAGGCGTCTCGAGTTCTCCATGTCTTCTTCTAGAAAAGACCCCATCACATTCCAGAGTGTGCCTACAAAGCAAAATAAAGGA**GAA**GG**CTC**TA**GA**
A(TaHSP16.8E1)CATGGTGGTTTCTTCCTGGGCC**GAA**AATA**GAA**A**GAA**AGG**GAA(TaHSP16.8E2)**GCTCACGGCCCAGCTGGAAAGTGATAAAGTACTACTGAACTCT
CGTCGACTGGAGAGACCTCGAGACGCTTCAGGAGCATCACCCGGCGATGCTATAAGTACATGGCCTCCCATCCCCCTCCATCCCAACTCAAGCCAGTGTCAACCAAAAG
CAGACAGCAACCCCAACCTACCTACGATCCAATACAGAATTTTCCTAGCGCAAGAGTCAAAGCAAGCAACACCGGCA**ATG**

TaHSP16.9b (assembled)
GACACAAGAATCAATACTCAAGCTGTAGACTAGTTTCTTGATTTACTTCATGATTGACATGTCAAAGTGCTTTGGTTTGCAACCAATGAGGAAGCCCACACTACTATCC
TGGAAGATGCAGCTATTACTCATTTCACAGTAGGTTGACAATATGCAAATGATTTCCTAGGGGTTTCAGACCTGCTATGTGCTTATGTATTGAAATGCAATGGCATGAT
GGAACGCCAGGCACACCACATGAGCTGAAATCAGTGCATAGCAATGGAACTTGAATTGAGTTTCAAAACAAAACAAAGCTTTAGTCTGAATCTCTGGAGCTGCGGCGTG
GCGGGGCCGCCGGCCCAAGATGACGCGGAAGGGACGGGGTGGGGCTGCGGGGGAAGGGATGAGGTCGACGGAGGCTGGTCGGCGAGGAGGTGATCCACGACGCGCAAGG
GAACCAGGTAGTGGCAAAAATCGAACCTTGCCGCTTGCTCTGTTTTGGCAGGGGAACGAGGCGCTGGCGTGGGGGGACGAGGCGGCGACGTGGTGCGGCGGAGAGGTGG
GTAAGGGAGCATGGTGGCATTGGGCGTGGGACGGATGGGGAGGCAGCGCCGTCGCCGGCTGGGGGGAGCGATGGAGAAAGCGAGGAAGGAGACGGGTGGACTGCGGGTT
TG**GTC**GG**GAA**AA**TTC**GAA**GGA(TaHSP16.9bE2)**CTAAAATGCAAAAAACGCCCTTGCGACAAGAATTACGAACGGAGGGAGTAAAAACAAACCCGAAA**GAA**ACCGT**TT**
CAAT**GAA(TaHSP16.9bE1)**AAAAAGCCCAACACGAGTCGCGTAGCGACTTTGGAACGCTCCACGCTAGAAAGTGCCCTCGGCGGAGGTTGTCGTTAGTTGCTCACCGA
AGTTGTCTGGTTTCCATGTCGTCTTCTAGAATAGTGGCCCAGGTGCTGGTACAATTATTAACAAGAAATAGAGAGAAGGCCCACTAACGGCAAGTAGGAGACTCTAGAA
GACGGTAGGCCCTTCAAGGGCTGATAACCGAAGGAAGAAAGCAAACTCTTGCCCATTGGAGAGTTCTCGAGATGCTTCAAGACCAGCAGCCGACGACGCTATAAGTACC
TGGCCTCAGCCCTATCGCCCTCCATCCAACTCAAATCAGTGTCAAGCAAAAGCACACAACATCCTAGTCCTACCTACGATCTACAGAATTTTCCGAGCGCACAAGCCGA
AGCAATCAACACCGACG**ATG**

TaHSP17 (KF208539)
GAGAAGAGGAGTGGTACACGTGCTGTCACCGCCCTAGAATATTATAGTGTAGTTAGTTGTAATAGTTGCACTAGAAGCATGCATGCATCATGTTTAAATTCAAGAATTT
TTTTGAAGGAAATTAATAGCCTCAAACTTTAATAAAAGGGCAAAAACCCAAATCCACACACTCAATGT**TTC**CA**AAA**TG**TTC(TaHSP17E1 in minus-strand)**
TTATTAAATGTTGATATTTCTGGCAAGAGTTATGACTCCAACCAAATTTTGTGAACATTTTTAAGAATTGTAGTTGGACACTAAATTTAAATCACTATTGTATTTGGAT
CTTTTCTCTAGCATTATATGGAGATTTCCAAAGGAAAAATGGAGATGCGAGAGCCACGCGACCGCGATGAGGCCTACACTACACATCAACACTACTTTGATCAAAACAA
CTTCAAGACACCAAAACCACACGGTATTTAGAATTGACGTGCATGGTTACACGATTCAAGTGTTTTGAGACCAACTTTTCTGCTAGGAACAACTCTCCATCGAGGATGA
CATTTTTAGCTGTCGTCGCTTTTAATCAAGCGGTATGTACATACTACTATTGCTTAAGTTAAACCTGGACAAAAGATTTGACTCTGTCTTATATCAGGTTCCTCAAAAA
GTCAAGTAATTGGATTGTACTCCCTCTGTTTCATAATATAAAAATGTTTTTCAAGCTAACATAGCTTTAAAAATGTTCTTATATTATGAGACGGATGGAGTAGTTGATT
GGCGTTCTTCTCGGTTGGTCTCAGCTCCTGTGAGTAGTTGATTGTGCGACCGTGCGAGTCGCTTGCTTGGTGCGCTCCTGAGGCATCTCGAGTTCTCCATGTCGTATCC
TAGAAAACGCCCCATCACGTTCCAGAGTACTCGCCGAGGTGCTGCAGCTGTACAAAGAAAATAATGGAGAAGGCCCACCAACCACAAAGAAGGAGAGTAGTCTAGAATA
CGGTAGCACCTTCATGGGCCGAAAATAGAAACAAGGAGAAGCTCGTGGAGCCAGCTGAAAAGCGAAACGTAAGCAAACGCTCGTCGATTGCAGAGTGCTTGAGATGCTT
CAAGAGCAGCACCTACGAGGCTATAAGTAGCTGGCCTGAGTCCCATCGCCCTCCATCCCAACTCAAATCAGCGTCAAGCAAAAGCAGACAGCATCCTACCTGCGGTCCG
ATACAGATTTTCCCCCGAGCTACAACCCAAAGCAACATCGACA**ATG**

TaHSP17.3 (KJ685919)
AGAAGTTAAGTATCACCACGTATATGTATAGAAAAAATGCATCCTACCATCTGGGGGTAATTTTTTTTGAGGAACCATCTGGGGGTAGTTACCCCACATGTTTCACATA
ATATCATGTAGTAGTATATATAATACTGTATTATTTTAAATATGTTCATATACTATATTTAAGATATTTCAAAGCAAGTAAATGAAAAAACTGCATATGAGTCCATATT
TTTTGTTATATTTGTGAAATACATATATATTTGTACGTAAAAAAGAGTATGCTTAAAATATTATACATAGTACATAAAAAGTAGTATATATACTATCCAAACATTGTTA
TATATTACATATTACACGTACATACTGTCGATTTTGACAGATACTATATACAACATACAAAACGCAAGTGGTGGTAATTATCACTGGTGATAGGAAACACTTGTGTTTC
TCTCAAGAAAAGCAGAGCTATTTTGTTTCTCTGTAGAGACGGCAATCTTAGCCACATGCTTTGCTTGGGATCTGGTCCCCTTTCTTTCCCTCTAAACATTTCTATCATT
TTACATGTCTGAAATTTATTTCCTCATATGGCCTAAATTAGAAGAAATATAAACATTTCGAAATATAAACAAAAATAAAAATATAGAGGAAAATACAAACTCAAGGCAA
ACACATGGCCTAACTTGGTTGGTTCACCGGAATGAGTTAGTGCGAATTCTTCATTTTTATTGTTTGAACCAAGCTACTGTAGCTAACCGAAATGAGTTAGTGCGAATTA
TTTTTTTAAGAGAACATTTTTTAAATAAAGTTTTAAAAAATTGAAATCTCAACATATTTTGAGAAGTGCACACATTTTTTGAATTTATGAA**GAA**AC**TTC**TT**AAA(TaHS**
P17.3E2)ATACAAACAATTTTTAAAGCCAAGAATATTTTTTAAATTCCCAAATTGTTTTGAGTTTGTGAACAAAATTTGAAAGAGGGGGGAATATTTTTTGAAGCTGTG
AACATATTTTCAATTTGTCAACAAATTTTAAAAAGGGGGAACAAATTTTGAAACCATGAACAAATTTATGAAAATATTTTTAAAAAGGAAACATTTTTTTAAATTCCTA
ATAATCTTTAAAATGAGAATAGCTTTTGTAATGCAACACTTTTTCCTGAATTTATGCACAAATTTTGAAAACATAAACATTTTTATGTATTTTTTTGAAATTTAGGAAC
ATTTTTATGAAAAGAAATATAAACTTGAAAAGGAAAAAGGGAAACAAGTTGGCCGGCCGATTTACCTGGTCGCTCGTTGTGCTCTGTGTGCTTTTGTGACAATCTGCCG
CAGAAAGCGTCAAATAGAAATGCGACGATCTATAGACGCGCCCAGAACTATCTAGTTTATGATAGACCTGAGTAGCAGGGTCCAGAGCGAATGGTGGAAGCATTCCAGG
CCCAGCTACACGATTCTTGCTTCTACCTGTACGCGCTCGAACAAACGA**GAA**CT**CTC**AA**GAA(TaHSP17.3E1)**CCTCATATCCATTGCACGAACGCCCCACTATAAATT
CCCACCGCCTGCATTGGCTCCGTCCCAAGACCGGCAGAGAAAAACCATCGAAAGCTTCTTTGATCTCGATCGCACAAGCCATTCGCGCTCACGCACCACTCCACCAGTA
AACAAAAGATTCATCAGCAGCTTCTCGCCAGCCCGCTGACG**ATG**

TaHSP62.4 (assembled)
TAATCATGATTACACGGCCAGTCACACATGATTAATTGATCCAACCAAAAACCACGTAACAGACGCGCGTCAGTGCGTGACTGTTGGCTTCTATCAACGTTTTATTGCC
ATGTTTTAATCTGCCGCATTGATCTCATAATACAGGAAATAAAAGGAAAAAGAAACGATACCACGTACGTAAAGAAGCCAGCCACAGAAAATTTGGCTAACGCTTAATT
AGCCGATTAATCCGCCACAGTGCCACTTATCTTCTGCGCTTAAACAGGAAACAATGCACACGCACAGAAAGAGCCCCGTTGCCAAAATTTTGGCGCTCGTTTTCTGCGC
CCTTGCCACGCCAATAAATTGGCGAATATAGAACGGGGGCGTTCAGGGCGAGCCGGGCGAGCCAAAATTTTGGCCTCGATCCAAACTACAGCCAAATCCCGTCGGTGTG
CCAATTTTTTGGCTGGGCAAGTGTTGGTAGTAAACCAAACAGCCCCTTACCCTTCCCCACTCCTTCAGCGGCGTCTCCCCTG**TTC**GC**GAC**GC**TTC**CG**GAA(TaHSP62.4**
E1)CCTCGGCGGCGGCCCCGCCTGAGGCGCCCGCGTGTCGTGCTCTCTCCCGGCCTCTCCAGAAAATGCCTTTCCACCTTCTTCTACCTCCCCCACCCCCTCCTACAGC
CCACTTCCTCTCAACCCATTTCGCGCACACCCTCAGTTCGTTTTGCGCTACCGCGGAGAAGCATCGACGAGCAAGGCCGGCGAAGAGAGTCCCCACCGCGCATCCCTCG
CCCGTTCCGGTAAGGCATCTCCGAACCCTACGCATGCCTAATTTGATCCGTGCCTGATTTCTTTAGATGGACGCGTCGTGGCTCGTTGATCCGAGTACGCATTCCGCTG
AGTTCAGTATTAGGTGCCTATGTGCTTGAGTTATATCTGCAATTGGGTAGTTCACTAGTACGTGCGAGCGCTAAGGTTTACTGGAGAACTTTGATGTGCAATTTGTTTA

GGGTGGGAAACATATCGCTTAATGCAGTGATGTTTGGTTTTGTTGCGGTTCAGGTACTAAAATTTGCCTTCTATGCAAGGTGGAGAAACCAGCAGCCGTGACATTGTTA
CTCATGTGCGTAGTTGTGCCTGCTTGTTTGAAACCCAGGCATTGCTAnnn(a large-
intron)nnnTACTCATCAGCATGCATATTCTAGACGTTTTGCATGCAATTATTAGTTTTGAATGTCCTTGACCTATCGATCTAGCACTGCATATTCAGACGTTTTGCG
TGCAATTATTAGTTTTGAATGTCTGAATACTCAGCAGCAGACGGTATAATTTACTGCTATCACTGTTTAAAGAATCTGGTTTTGGACAACACTTTAAAAGAATCTAACA
ACGCCTAACTGGTTTGACTGTATTTTGCAGG**ATG**

TaHSP90.1-A1 (KF208540)
GGAGACATTTTCTGAACTGGCGAAGTTTTTTTGAAATGTGGGAACAAATTTTGAAATTCACGAACATATTTTTTTCCTTTTTTAGCATTTTTTAAAAATTTCATCAACA
CTAATTCGTGTTTCTACTGAAATCAATGAAGATCACGAACATTTAAATGAACACTAATTCGAATCAATGAACATTAAGAAATATCATGAACAATAATTCATGTTTCTAT
TGAAATCAATGAACATCACGAACCTTTATAAAAGCATCATGAACACTTTTTTGTGTTTCTATTGAAATCGATGAACATCACGAACAATAATGTAGAAAGATGTTAAAGT
AAAAACGTTCAAGTCATCTTGTCCTGACAAAATGTTGACACGTGTATTAAAAATGTTCAAATTGTCTTAAAGAATGTACTTAAATGGTGTGTTAAGAATGTTCATCGTG
AACTTATAATGTGTTCATCATGTCTCAAAAAATTTTCATGAACTTAACGGATGCTTGTAATGTACGAAAACATGTTAATCTCATATTAAAATGGTCCATGTGTATATAG
AAAATGCATGCCATATTAAAAATAGTTTCATCACGCATTGTAAAAACGCTTGTCTTATGTTTAACATAAGTTACCCATGTATTTTACAAGATGCTCGCGTAGAAAGCGG
CAAGAAATTAAAGGAAGCCCCGATAGAAACCCGAACTTACGAGGTCATTTGCCGAGTTCTCTTTGGCATTGTTCTCTCCGCATCCAAGAGCGCCCCGCCTTCTCAACGC
CCGGGCATCCATCCAATGGTTTTATTTTTTATTTTTGAGGGGTGGGCATCCGTCCAATGATGATTCTTGATGGCGTCGAACTTGAGCGAACGCCCACACAATCTCAATT
TGACACAGCAACACACACACCCTCTACCACCTATTCACTTTTGCTTTCTGGTTTGGGCATCCTGGGCCGGCCCATACGCGTGCGTACAAGCGAGATATAGCAGCCTCC
CCATTTCTGCTAAGGTCCATTGTAGTATGTAGAAGCTTCGGGACAAGGCTCGGGCAGGCCAAGGCGACCGCCTCGCCGCTCTCCGCACCAACGGAGCGGGAGGGGGGAG
AGACCACAA**GAA**GC**TTC**GG**GAA**(**TaHSP90.1E1**)CGCCCGCGAACGCGCGCCGGAGCTCGTGCCGGTAATCCGTGGAAGGCACCAGAACACCTGCGAAGGCCCCTAGCG
ATCCCATCTCCGGGGTATAAATCCTCCCGCCCGCCACCTGTTCGTCAATCTGTCTGTCCCCAAACCTCTTCCCGCAGCGCGACAAGCAAGCGATCAGAGCAATCAACGC
AGCGAATCGGCCGAGGAATCAGCAGCCCACCCAGCCATCTTCCCCCAATTTCCCTTCGAGAAGTGGCAGCGGAGACGGCA**ATG**

TaHSP101 (assembled)
CTATGCGTCGGCCGGCTGATATTTAGAAAACATCCGCCGCCTCGGTAACTATAGGATCACGAGCTCGACAACCGTCCAATCTTCTTTCCCGATGTTTTTTCCGTGCATC
AATCTTTTCAACAAGGCCCGATCCTTGTTGTGCTCTTGCCGAACTGTTTTTTCTTTGAAACAAGGCCTATATTTCAGAAACAAGACCCCTGTTACAGAAAAAAAACTTC
GTTGTTGCCTCCTTTGTGTTGTGTTCTCCCTCTACAACAAGAAGCCTGTTGTAGAAAAAAAATCCCCTTGCAATTAGTGATTGTTCGCCACCACCATTTGCAACAAAGT
CGTTGTTGCGGGAACTCGTCTCGCAACACCACGCTAGCGGTGAGGGCCGCGGTGAACGTTGCACGGTAGCTGGGTAAGGTCCCCGGTGCGACGTGGGGATGGGGCAGCG
AGCGAGGGCGCGATAAACAACACAAGCTTCACCCCTCTCCCCAAGCTCGCAGGAGTTTTACAACAGCTAGGTTTCGCTATGGGTGCTCTCTCTTCTCACGAATGGCACA
TGATTTGGATGCGTGCTTGGGGGATTTACATCCATGAAAGGGGCCTGGAGGTAGGAGACGAGGTTCAAGGAAAAATATGTGGTTTTGCTCTTTTCGTCTGGGATAAGAA
GATAAGAATGGGAGAGCGGCTGCGGGTCCTATGTAGGCACGTGCCGTACAATCGGATTGGGTTTTGCAACACGGTCCCTGCCGCAGAAGCAACCTTCACAACAAGAGCC
ATGTTACAAAGCTCATGCGTATAATAAGAATTGTGTGCCGCACGATTAAAAGTTAATCTGACGATTGCAGAGGAGGCCGTCGGATGATACCAAGCATTTTCCTTCAGAA
ATTGTACTTCATGTCCATAGTTTTTTAAGGGAATTTCATGTCCATAGTTGCAGGGCACAGAGGAAGGCATGAAATCGGAACCAACGGTCGGGATACTGTCTCGTGCGAT
AGGAGATACAGTAATGCGCACGGTTTCGGGTTTTCCAAATAAAACGGTTCCACACTA**GAA**TG**TTC**TG**GGA**CA**TTC**(**TaHSP101E1 in minus-strand**)
TTGTCCCCCTAACTCGCCGCCTTCCCGCCGCTTCCACGCCGTTCTAGACCTCCGGCGAATCCTCCCCTCCCGCCTCTTATTACCCGTCTCATCTCCGATCCCCCCAGCA
ATTTCCGACGAGAAGAAGACACCAAGGCGAATCATCCAACCAACGAACTCCCCCCCAATCACCATTGCCAGTTCCGTTGCGGTTAATCCGTAGCTCGCAGAAGAGGAAG
ACATG

TaRCAL (KJ685916)
CGTAAGCAAGCCTCCCACTTATGATTATCCCCTCTCGCAAGCATCCGCAATTAAGAAAGAAGAATTAAGATAAATCTAATCATAGCATGGAATGATTTCTCTCTCGTCT
ACCTCAACATTTATTTTATACGGGACTCTTAAGATAGCATCATTGTACACGCCCTTAAGTTGTACACTCAAAAAATAGAATGCGATTCAGTTGTATCTATAAAATTAAT
TTCACAGTTGTCCAAATAAATAAGCAAGGAAATGAAAAATAACCATAGAGGCTTTTTAGATAAGATGGAAATTTTAGATCCTGTGGTGATGTGCAACCTAGTAGATACA
AAACAAAAAAGCTAG**TAC**AT**GAA**GT**TTC**CT**GAC**(TaRCA-LE1)
AGTAACCCGGGCCCTACCGGAGGGAATGAGATTAGTACTTTGTGGTGGTGTAAAGCCAGTCTAGACGTTTCATGCAAAATGCAAAAAGAAAATGGAACGAACAAGATGT
GATTTTTATGGTGAAGGATGAAGGCATCATTGTTTCTACGGCGGCGCACGCTGATTTAATAATGCATGCATACCCGCAAAAAGATGCTCCGGGCCCGTAACCCACAAGG
AACCGAAGGCAGGCAGCTTAGATTTCTTTTAGGCCGTTGGATGCGCCGATCAGGAGGCTGACAAGCAGCCACGTCGACGCCTTTGATGGACTGAGCCAGGGTAGCCAGG
AAGCCAATCCTCAGCGTGTGGCCCCTGGGCGATGCCCAGCCCCATCCACACAATTAACATCTCATCATCCACTCCACTTGGCTCGCCTCACTACTTATCGCAGCCATCG
CGCTAAGCAAAGCCAATTAGAGCCTAACAATCACAGCTTATCTCGCCTGACTGCGTGATTACGCAGGGAAGCTCTAGAATAGTCGCGCACGCTGTAGGTCACTCCAGTC
GTGTTGGACGTTTGCACCATGGCCGTCTGTGCGTGCACCTATATAAGCCGCCATAGCCAAGCTGCACCGATCGGCATCGCATCTGTACTTGCTAGCCAGCGCACCCTCT
CTTTTATCCACATCAGCTCGGTCGATCACTGACCATCTCAGTCGAGCTGCATCTGCAGGTGTACTAGTAGTACGCCTAAGCTGAGCTTGCATATACCACTGCACGTACG
CACATCAAATTAATCTGAAACTAATTAAGTTATCAGGTGCGGGGAGAGAACAATCGACACG**ATG**

TaRof1 (KJ685917)
GGAGAAGTGATGACGATAACCTCGGTGTGCGATCTTGGTGAGGCCCTCTATGTGATGTGTGCATGGGCTATTGAGCGAGTGCCGCTCAACGGTTTGTGACGATTTTCAC
TCGGTATTCCATTAATTAATTGGGAACTCTCTTCTACTTAATCGAGGGATGAGGCAAATCTTTTGCCTCTTTTTTTCTTAGAAAAAACCCCGCCATCGAGTATGTAGTG
ATTGCTTGCACCAGTGACTTGATGGAAGTTTTCTTGCAACTCCTAGTCCCAGCTATGCATTTCTCTACCCAAACTTGAAAGCTTTTTCAAATTCTGTTTTTCTTCAAAT
AGTATAAGCGAAGACAGCGTCTTGGGATCGGCTGACATCCGAGGGAAGGCCCAACCCAACTTCTTCACACTGTTTGCTGTGGCCCAGTTGTCAAAATTATCATCATCTT
CGAGGAACCGTCTTTAGGTAGTCCAGGCCCACAAAGTGAAACCAAGGACGACTCGGTCCTTCTCCCCTGCATCTCTGGAAGCATCCACTGCCTTCCAAAGCTCTCGCGC
CCCCGCTCCCCACCCACCCTGATCTGGAAACATCTCCCAATCCAACCCTCTCTCACAGGAACAG**GAA**CC**CTC**TA**GAA**(**TaRof1E1**)CATCACATCGTTCAACGCAACGA
TCTCTGCTCCGCCATATAAGAAGCCAGCGATCCGAGACGCGCCTCGCTCCGGCTGCAAACAACATCCAGAACGCACAGACACACAGATTACGAGCGAGCGCGCACCCGT
TAATCGCTTCATCTTTCCATCCCACCTAGAGAGCCTTTCGCGTCCACGAACCTAATCAACA**ATG**

Supplementary Figure S1. Analysed promoter sequences of heat upregulated genes from *T. aestivum*.
The core motifs of putative heat shock elements are in red. The translation initiation codon ATG is in bold.
TaHSP16.9b, *TaHSP62.4* and *TaHSP101* promoter sequences assembled from sequence databases as follows:
TaHSP16.9b promoter was assembled from the wheat genome sequences at http://www.cerealsdb.uk.net/CerealsDB/Documents/DOC_search_reads.php, based on its ESTs from NCBI (GenBank accession #**:** HX017006, CJ618927 and CJ722864).
TaHSP62.4 promoter was assembled from the wheat genome sequences, based on its ESTs (GenBank accession #**:** CJ725593, CJ636112, HX161644 and HX161669).
TaHSP101 promoter was assembled from the wheat genome sequences, based on its cDNA (GenBank accession #**:** AF174433).

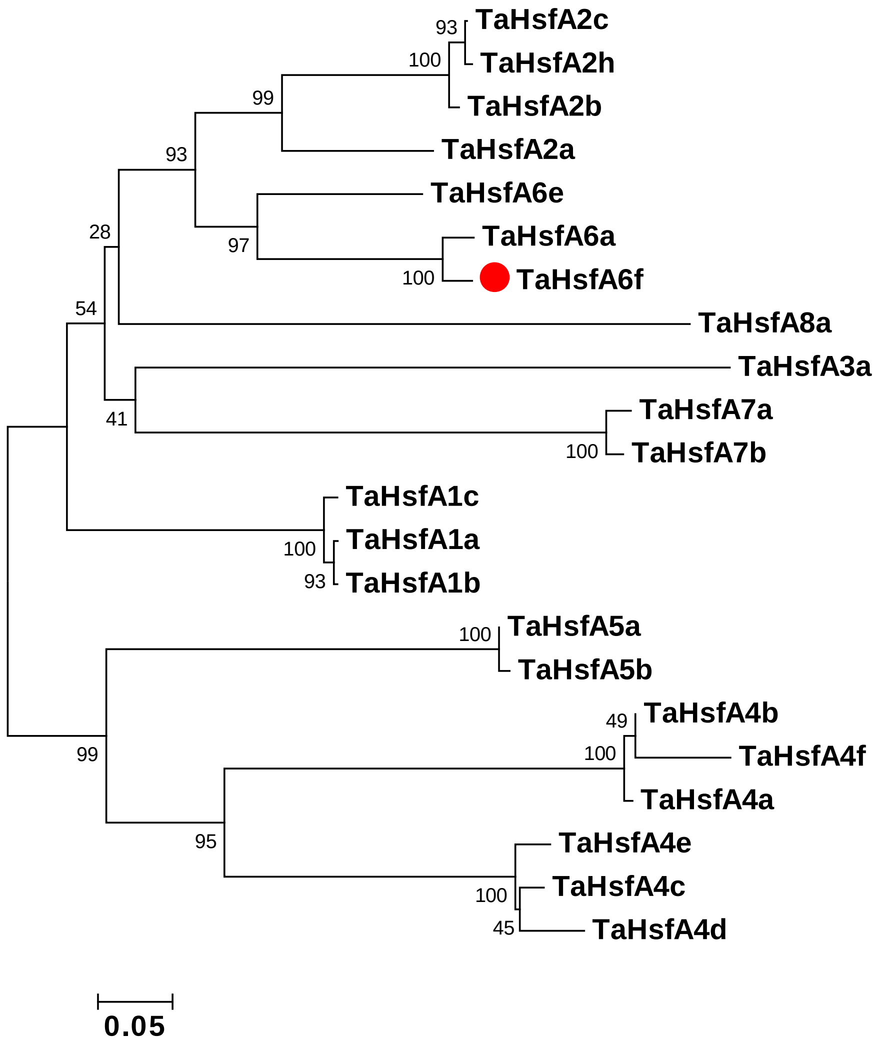

Supplementary Figure S2a. Neighbor-joining phylogenetic tree of wheat HsfA proteins.
The N-proximal regions (from the start of DNA-binding domain to the end of HR-A/B region) of HsfA proteins were used for construction of the phylogenetic tree using MEGA 6.0 program.
Un-rooted neighbor-joining analysis was performed with pairwise deletion and Possion correction.
Bootstrap values are indicated. TaHsfA6f is marked. The rest of HsfA proteins were reported previously (Xue et al., 2014). TaHsfA members that contain partial DNA-binding domain and HR-A/B sequences identified from the previous study are excluded from this analysis.

```
            1                                                                                                                          130
TaHsfA1a    MDGGVAAVASAAAAAAAASSTVTTAVAPPGAGAGAGAPPPFLMKTYDMVDDPATDAVVSWGP-ANNSFIVWNTPEFARDLLPKYFKHNNF
TaHsfA1b    MDGGVAAVAAAAAAA----STVTTAVAPPGA-AGAGAPPPFLMKTYDMVDDPATDAVVSWGP-ANNSFIVWNTPEFARDLLPKYFKHNNF
TaHsfA3a    MDHTHTGINTTPAAVTASASMDAALLLEPKLEMMQQQPSPAGHYAALDHHLIPPPPALVVPCEPPRPLEALLQGPQLPPFLSKTYDLVSEPQLDGVISWGP-AGNSFVVWNPSTFARDVLPHNFKHNNF
TaHsfA4a    MEGSGGSSSLPPFLTKTYEMVDEPATDAVVAWTP-SGTSFVVLSQADFCRDLLPKYFKHNNF
TaHsfA4b    MEGSGGSASLPPFLTKTYEMVDEPATDAVVAWTP-SGTSFVVLSQADFCRDLLPKYFKHNNF
TaHsfA4c    MDMDMDMEG-GSQSQPQGASLSPAPFLIKTYEMVEDPATSRVVSWGP-GGASFVVWNPPDFSRDLLPKYFKHNNF
TaHsfA4d    MDIDMEG-GSQPQPQGASLSPAPFLIKTYEMVEDPATNRVVSWGP-GGASFVVWNPPDFSRDLLPKYFKHNNF
TaHsfA4e    MEMDMEG-GSQPQPQGASLSPAPFLIKTYEMVEDPATSRVVSWGP-GGASFVVWNPPDFSRDLLPKYFKHNNF
TaHsfA5a    MEGSGAVGARGGGGGGPAPFLLKTYEMVDDPATDAVVSWSDASDTSFVVWNSPEFAARLLPAYFKHSNF
TaHsfA5b    MEGSGTAGARGGGGGGPAPFLLKTYEMVDDPSTDAVVSWSDASDTSFVVWNSPEFAARLLPAYFKHSNF
TaHsfA2a    MSHRMMMSPVKVEGRRCPDPDPAAAAGGGAP----------RPM-DGLGDAGPTPFLAKTYDMVDDPATDAVVSWTA-TSNSFVVWDPHLFATLLLPSYFKHSNF
TaHsfA2b    MDPVPGLVKEEEEE-GAHGRGDSPAVGA---AP----------RPM-EGLHDAGPPPFLTKTYDMVDDPNTDSIMSWSA-GNNSFVVWDPHAFATVLLPRHFKHSNF
TaHsfA2c    MDPVPSLVKEEEEE-GAHGRGDSPGARA---AP----------RPM-EGLHDAGPPPFLTKTYDMVDDPNTDSIMSWSA-GNNSFVVWDPHAFATVLLPRHFKHSNF
TaHsfA2h    MDPVPSLVKEEEEEEGAHGRGDSPGARA---AP----------RPM-EGLHDAGPPPFLTKTYDMVDDPNTDSIMSWSS-GNNSFVVWDPHAFATVLLPRHFKHSNF
TaHsfA6a    MDRVLLPVRVKEEWPPPPPEEEEELEHGGLAP----------RPM-EGLHETGPPPFLTKTFDLVADPATDGVVSWGR-AGNSFVVWDPHLFAAVLLPRFFKHSNF
TaHsfA6f    MDRVLLPVRVKEEWPLLLPEEEEELEHGGLAP----------RPM-EGLHETGPPPFLTKTFDLVADPATDGVVSWGR-AGNSFVVWDPHLFAAVLLPRFFKHSNF
TaHsfA6e    MDAMPPEGIVKEEEVLLHEEHTHPQAGPQQQQQRQDGAL-P----------RPM-EGLHEAGPPPFLTKTYDLVEDPATDQVVSWGR-AGNTFVVWDPHVFAEALLPRLFKHSNF
TaHsfA8a    MGSSKGSPPPSAAGPSSAGNAP----------APVGPAPRPPEVAPFLTKVYDMVSDAATDKVMSWTD-AGNSFVIWDAHAFERDLLSRHFKHRNF
TaHsfA7a    MDYAAAVTMDIKQEEPEMVVLDDDDDGDAGCCLAP----------TPL-DLAAAAAVAPFLAKTFDMVEDPATDAVVSWGA-ARNSFVVWDPHAFAARLLPRHFKHANF
TaHsfA7b    MDSAAAVTMDVKQEEPEMVVLDDDD-GDAGCCLAP----------TPL-DLSAAAAVPPFLAKTFDMVEDPATDAVVSWGA-ARNSFVVWDPHAFAARLLPLHFKHANF
Consensus   .......................................ap...........p.........ppPFL.KT%#$V.#PaTD.!vSWg. agnSFVVW#ph.Fa..LLP..FKH.NF
            131                                                                                                                        260
TaHsfA1a    SSFVRQLNTY--GFRKVDPDKWEFANEGFLRGQKHLLKTINRRKPSHANNQVQVPQQQQHHQQQHQQQPQLQNAPMPSCVE--VGKFGMEEEIEMLKRDKNVLMQELVRLRQQQQTTDHQLQTLGKRLHG
TaHsfA1b    SSFVRQLNTY--GFRKVDPDKWEFANEGFLRGQKHLLKTINRRKPSHANNQVQVPQQQP--QQQHQQQPQLQNAPMPSCVE--VGKFGMEEEIEMLKRDKNVLMQELVRLRQQQQTTDHQLQTLGKRLHG
TaHsfA3a    SSFVRQLNTY--GFRKVHADRWEFAHEGFLRGSKHLLKTIVRRRSSPT-------------QQSSLQPGSSIFRKIQSGS--SGESTVDPELRSLRREKNALLQEVARLKEEHNQTIEHMNALNQRLET
TaHsfA4a    SSFVRQLNTY--GFRKVDPEQWEFANEEFIRDQRHRLKNIHRRKPIFS---------------HSSHTQGAG----PLAD--SERRDYEEEIERLKCDNASLKLQLERK---KTDMESKMKVLEDKLFA
TaHsfA4b    SSFVRQLNTY--GFRKVDPEQWEFANEEFIRDQRHRLKNIHRRKPIFS---------------HSSHTQGAG----PLAD--SERRDYDEEIERLKCDNASLKLQLERK---KTDMESKMKALEDKLFA
TaHsfA4c    SSFIRQLNTY--GFRKIDPERWEFANDDFIRGHMHLLKNIHRRKPVHS---------------HSPQNQVNG----PLAE--SERREYEEEISRLKHENSVLVAELQRQAQQQCGLSWLMQSLEERLLA
TaHsfA4d    SSFIRQLNTY--GFRKIDPERWEFANDDFIRGHMHLLKNIHRRKPVHS---------------HSPQTQVNG----PLAE--AERREYEEDISRLKHENSVLVAELQRQARQQCGLSWLMQSLEDRLMA
TaHsfA4e    SSFIRQLNTY--GFRKIDPELWEFANDDFIRGHMHLLKNIHRRKPVHS---------------HSPQNQVNG----PLAE--SERREYEEEISRLKHENGLLVAELQRQAQQQCGLSWLMQSLEDRLTA
TaHsfA5a    SSFIRQLNTY--GFRKIDPERWEFGNEYFVKGQKHLLKNIYRRKPIHS---------------HSHQP---G----ALPD--NERSFFEDEIDRLAREKANLQAELWKSKQQESGTMFQIEALERRAVD
TaHsfA5b    SSFIRQLNTY--GFRKIDPERWEFGNEYFVKGQKHLLKNIYRRKPIHS---------------HSHQP---G----ALPD--NERSFFEDEIDRLAREKANLQAELWKSKQQESGTMFQIEALERRAVD
TaHsfA2a    SSFVRQLNTY--GFRKVDPDRWEFANEGFLRGQRHLLRNIKRRKP-THG-------------SQNQQSL------GSYLE-VGNFG-HDVEIDHLKRDRQLLMAEVVKLRQEQQNTRSDLQAMEKRLQG
TaHsfA2b    SSFVRQLNTY--GFRKVDPDRWEFANEGFLRGQRHLLKNIRRRKPPAHT-------------ASNQQSL------GSYLE-VGHFG-YDAEIDRLKRDKQLLMAEVVKLRQEQQNTKARLKAMEDRLRG
TaHsfA2c    SSFVRQLNTY--GFRKVDPDRWEFANEGFLRGQRHLLKNIRRRKPPAHT-------------ASNQQSL------GSYLE-VGHFG-YDAEIDRLKRDKQLLMAEVVKLRQEQQNMKVHLKAMEDRLRG
TaHsfA2h    SSFVRQLNTY--GFRKVDPDRWEFANEGFLRGQRHLLKNIRRRKPPAHT-------------ASNQQSL------GSYLE-VGHFG-YDAEIDRLKRDKQLLMAEVVKLRQEQQNMKVHLKAMEDRLRG
TaHsfA6a    SSFVRQLNTY--GFRKIDPDRWEFANEGFIRGQRQLLKMIKRRRPWSYL------------PSPQHQAL------GSCLE-VGQFG-LDDEIEVLKRDKNALLEEVVKLRQEQQSSRADMRAMEERLHR
TaHsfA6f    SSFVRQLNTY--GFRKIDPDRWEFANEGFIRGQRQLLKMIKRRRSLSYL------------PSSQQQAL------GSCLE-VGQFG-FDEEIEVLKRDKNALLAEVVKLRQEQQSSRADMRAMEERLHR
TaHsfA6e    SSFVRQLNTY--GFRKVDPDRWEFANEGFLRGQRHLLKMIKRRKPPSNA------------PSSQQQAL------TSCLE-VGEFG-FEEEIDRLKRDKNLLITEVVKLRQEQQATKDNVQAMEGRLRA
TaHsfA8a    SSFIRQLNTY--GFRKVDPDRWEWANEGFLRGQKHLLKIIKRKKRPQEA------------GRELEKAPVKAAPGTENIE-IGRYGGLVKEVETLKRDKSLLMQQLVDLRHYQQSSNLEVQSLIQRLQV
TaHsfA7a    SSFLRQLNTY--GFRKVNPDRWEFANTGFLGGQRHLLAGIRRRRGADTG------------RRPAAALS----PSS-CAESAGGFGPVEGELEQLRRDREALKRELAGLKRQQEEARATLLDMERRVQG
TaHsfA7b    SSFLRQLNTYVSGFRKVNPDRWEFANTGFLGGQRHLLAGIRRRRGADTG------------RRPAAALS----PSSSCAEGAGGFGPVEGELERLRRDREALKRELAGLKRQQEEARATLLDMERRVEG
Consensus   SSFvRQLNTY  GFRK!#P#rWEFANegFlrGqrhLLk.I.RRkp....              .....q.........s..#..g..g..#.Ei..Lkr#k..L..#lv.lrq.qq......a$e.Rl..
            261                                                                                                                        390
TaHsfA1a    MEQRQQQMMSFLAKAMQSPGFLAQFVQQNENSKRRIVAANKKRRLPKQDDGLNPESALLDGQIIKYQPMINEAAKAMLRKILQQDTSPHRFESMGNSDNLLLENCMPSAQTFDSSSSTRNSAVTLAEVPG
TaHsfA1b    MEQRQQQMMSFLAKAMQSPGFLAQFVQQNENSKRRIVAANKKRRLPKQDDGLNSESALLDGQIIKYQPMINEAAKAMLRKILQQDTSPHRFESMGNSDNLLLENCMPSAQTFDSSSSTRNSAVTLAEVPG
TaHsfA3a    AEDRQKQVVSFLAKLLQNPDFLRQLKMHRERR-----------------DGI--DSARVKRKFLKHVPHGSRDSG--------DSSSPRTAESTCSPAHPVAHDAIADLQSFLLEDTDLSDGM----MPG
TaHsfA4a    IEGQQKNLISYVREIVNA------PGFISS--LI-----------EQSDHHGKKRRLPKPISFHEDASTQGNQ---IMHCDMVNSPTHELFRAS---FDKMESSLNSLENFFKEASEAFGND----VSY
TaHsfA4b    IEDQQKNLISYVREIVNA------PGFFSS--FV-----------EQSDHQGKKRRLPKPISFHEDTSTQGNQ---IMHCDLVNSPTHELFRAS---FDKMESSLNSLENFFKEASEAFGND----VSY
TaHsfA4c    MEQRQADVVASVRDIQQRRRGDVHPGQQTM-LEL-----------EPTDHFSKKRRVPTIGFFAEEPAAAAEE-----HLRATGGETPGMVMVNAEPFEKMELALVSMEKLVQRAGNYAPS-----EDM
TaHsfA4d    MERRQADVVASVRDTLQRRRGGVHPGQQTM-LEL-----------EPTDHFSKKRRVPRLGFFVEEPAAAAAEEQRVPHLRAMGGETPGMVMVNAEPFEKMELALVSMEKLVQRAGDYASS-----EDM
TaHsfA4e    MEQRQANVVASVRDTLQRRRGGVHPRQETMMLEL-----------EPTDHFSKKRRVPTIGFFVEEPA----EEQRVPHLRAMGGETPGMVMVNAEPFEKMELALVSMEKLVRRAGDYASSS----EDM
TaHsfA5a    MEQRQGKMIAFLQQASKNPHF-VSKLVKMAEASS-----------MFADALHKKRRLSGLDYAVEATETASFCDDHSATSRQEMGNLLNQHFSDKLKLGLCSAAAESNLVTLSTQSSHEDNG----SPH
TaHsfA5b    MEQRQGKMIAFLQQASKNPHF-VGKLVKMAEASS-----------MFADALHKKRRLSGLDYAVEATETASFCDDHSPTSRQETGNLLNQHFSDKLKLGLCSAAAESNLVTLSTQSSHEDNC----SPH
TaHsfA2a    TEQKQQQMMSFLARVMQNPMFIHHLLSQSEMRKE------------LEDAISNKRRRRIDQGPEAVDSMGTGSTLEQGSQVMFEQQEPVDSL-------------------------------------
TaHsfA2b    TEQKQQQMTSFMARILRNPEFLKQLIAKNGMRKE------------LHDAISKKRRRRIDGGPEAYAVGASSSSLEQELPVVFDSHGSVELLAEGSVPV--ELLADGIPPDLEGSVELLADEIPPGLEG
TaHsfA2c    TEQKQQQMTSFMARILRNPEFLKQLIAKNEMSKE------------LHDAISKKRRRRIDGGPEAYAVGASSSNLEQESPVVFDSHGSVELLAEGSVPVPVELLADGIPPDLEGSVALLDDGIPPDLEC
TaHsfA2h    TEQKQQQMTSFMARILRNPEFLKQLIAKNEMSKE------------LHDAISKKRRRRIDGGPEAYAVGASSSNLEQESPVVFDSHGSVELLAEGSVPVPVELLADGIPPDLEGSVALLDDGIPPDLEC
TaHsfA6a    VEQKQLQMMGFLARAIQNPDFFLQLIQQQDKLKD------------LEDAYSNKRRRSIDVMPFLGPEGA-SQSEPLESTFIFEDREFSE--------------------------------------
TaHsfA6f    VEQKQLQMMGFLARAIQNPDFFLQLIQQQDKLKD------------LEDGYPTKRRRPIDVMPFLGPEET-SQSEPLESTFIFEDREFSE--------------------------------------
TaHsfA6e    AEQRQAQMMGFLARAMRNPHFFQQLVQKQDKRKE------------LEDAISKKRRRPIDNAPFYGPGATTSQSEQLDSQFLFDSGVLTEPGMNG---------------------------------
TaHsfA8a    MEQNQKQMMALLSIVVQNPSLLNQLVQQQQQQRRTN---------WYEDGNKKRRFPALEQGPVTDYETSGAGTEIIQYRPPAPETSSQVIPDEAFLSA----------------------------
TaHsfA7a    TERRQEQCKAFLARAVRNPAFLANLARRNGLTAAAPA---------PAVDGKKKRRLLDVIPSPPPAEDGFTFEELALAAGVVEEAAAPTQGAGAG---------------------------------
TaHsfA7b    TERRQEQCKAFLARAVRSPAFLANLARRNGLSAAAPA---------SVIDGKKKRRLLNANSSPPPAEDGFTFEELALAAGVVEEAAAPTRS-------------------------------------
Consensus   .EqrQ.#m..fla...qnp.f..ql...........          ...D...kkrr......p.................................................................
            391                                                                                                                        520
TaHsfA1a    NSGMPYMPTSSGLSAICS--SSTPPEMQCPVLDSNSSTQLPTQLPNMSAASSIPKAMTPGLSDISIPGFPDLHDLITEDAINIPVENFAMPGPECIFPLPDEGSDDSVPMDPIDTDETDDTQKLPGIIDS
TaHsfA1b    NSGMPYMPTSSGLSAICS--SSTPPEMQCPVLDSNSSTQLPTQLPNMSAASSIPKAMTPGLSDISIPGFPDLHDLITEDAINIPVENYAMPGPECIFPLPDEGSDDSVPMDPIDTDETDDTQKLPGIIDS
TaHsfA3a    NFGLDGVEASEDIGALVQVFDQQDPGTGAELLGIPPVSGAAHCQDLTVGRSKGKNVMCPGGTDGTSSQADCLVPLPGNVGKLMDADGEQIWGADTFFQSSCSGTSQQTYVSDPYLIAIPDKHEKFWEVDF
TaHsfA4a    DGDVPGHSSAVV--LTELHSSGESEP---------HAQSPPSM----MHTCSAGVGDSHSSRDIAESAS-CPESSPLPEAHSRADSRSKVSEIDVNLEPAV-TETGPSRDQQPTQ------DPPADANDG
TaHsfA4b    DGDVPGHSSAVV--LTELHSSAESEPLHSSAESEPHEQSPPSM----MHTCSAGVGDSHSSRDIAESAS-CPESPPLPEAHSRADSRAKVSEIDVNLEPAV-TETGPSRDQQPTQ------DPPADANDG
TaHsfA4c    FSAATASASAAP--STDDPAHAGL-------------QEAP---------VETGVDLQPTSPELAEPPGYAVQSPMLLFPEIQEDKRNTTAEVDMSSEAST-TDTSQDETNAEAET----PHEQAVANDL
TaHsfA4d    YNAA---AAA-P--STDDPAHADL-------------QAAP---------V----NLQPSSPEIAESPGYAVQSPMLLFPEIHEDKHKTMAEVDMSSEAST-TDTSQDETTAEAEAETGVPHEPAVANDL
TaHsfA4e    YNAA---AAAAP--STDDPAHADL-------------QAAP---------VETGANLQPSSPELAESPGYAVQSPMLLFPEIHEDKRNTTAEVDMSSEAST-TDASQDETTAEAEAETGVPHEPAVANDL
TaHsfA5a    GRHPPVHDGMGTGCLPLVPQIMELSDTGTSICPSKSSFFAPAVNDEGLSPCHLSLTLASCSMDVDRGQASNADGSTTINEGSDNPPEATATAMGGNPKASVDAGTAEAPTPREDTWVAAEPAAPVKANDN
TaHsfA5b    GRHPPGHEGMGTGRLPLVPQMMELSDTGTSICPSKSSFFTPAVNDEGLSPCHLSLTLASCSMDVDRGQASNADGSTTVDEGSDNPPEATTAAMGVNQKASVDAGTAEALRPREDMRVATEPAAPVKANDN
TaHsfA2a    ---------------VNGVISDLESSSVDTKGAEVQQSVASSRSEQLRGRPSGELNDDFWEDLLHEGGLGEEASNLVVPDD-----MNLLAEKLD
TaHsfA2b    SVALLADGLEGSVELLVDGIPSDLDGSGIDSNGVTEPQDYGLGTCEAQQNRALGLFHDNFWEELLNKG-LSVENDEPVNVDG-----MDVLSEKMGYLIPNSPTLST
TaHsfA2c    SV-----------ELLVDGIPADLNGSGIDANGVTEPQDFGLGTCEAQQNRVPGLFHDNFWEELLNKG-LSGENDEPVNADG-----MDVLSEKMGYFIPNSPTLST
TaHsfA2h    SV-----------ELLVDGIPADLNGSGIDANGVTEPQDFGLGTCEAQQNRVPGLFHDNFWEGLLNKG-LSGENDEPVNADG-----MDVLSEKMGYFIPNSPTLST
TaHsfA6a    ---------------LENLAMNIQGIRKGMEGDRGGRDQGCGEAE---------LTDDFWEELLSE--GMRDEAEMVELERRRSRYVDA
TaHsfA6f    ---------------LENLAMNIQGIRKGMEHDRGGRNQGCGEAE---------LTDDFWEELLSE--GMRDEAEILELERRRSRYVDA
TaHsfA6e    ---------------MENLAQNIQELGQG-KTDEEKKDEANGQLD---------INNDFWAELFSDDFGDGDGSGLSELEGRRPEDIDELAQQLGYLSSTSPQ
TaHsfA8a    --------------TTQPISSPALNMPMDIDTQTTSDNLNTQGSSGDIFADMPALPDFDDMHLWFGEDGEPTLTIQDYDESPQSEQDCQMEAQHNYNNPQHADVITEA
TaHsfA7a    -----------------GVTTDMIWYELLEEGQAEIDVDVEDLVAAAGDMEPWGFGAEEVQDLMQQIDSFACSPSC
TaHsfA7b    -----------------GVTTDMIWYELLEEGQAEIDVEVEELVAAAGDMEPWEFGEEEVQDLMQQIDSFAGPPSC
Consensus   ..........................................................dl.......................................................................
            521                                                             586
TaHsfA1a    FWEQFLCASP-----LSIDNDEVDSGLLDTREAQEENGWTRTENLANLTEQMGLLSSNHRG
TaHsfA1b    FWEQFLCASP-----LSIDNDEVDSGLLDTREAQEENGWTRTENLANLTEQMGLLSSNHRG
TaHsfA3a    ---EALDDGD-----LHLDKCVIGDPALEQHRGNMKP
TaHsfA4a    FWQQFLTEQPGLSHAHQEAQSERRDREANQTTAGDRGSFWWGKSVEQMTEKLGHLTSAEKT
TaHsfA4b    FWQQFLTEQPGLSHAHQEAQSERRDREANQTTAGDRGSFWWGKSVEQMTEKLGHLTSAEKT
TaHsfA4c    FWERFLTDTP-----KPLAVEDSHESKDGVKMGLDCYWFGHRNNVDQITQQMGHLASAQYAHVCRQ
TaHsfA4d    FWERFLTDTP-----KPLAVEDRNESKDDVKTGLDCYWFGHRNNVEQITEQMGHLASAQKT
TaHsfA4e    FWERFLTDTP-----KPLAVEESHGPEDDVKTGLDCYWFGHRNNVDQITEQMGHLASAQKT
TaHsfA5a    FWEQFLTERPG--DASSGPRDPSMDKEQTEGGGKDDREVTEQLKL
TaHsfA5b    FWEQFLTERPG--DASSGPRDPSMDKEQTERGEKDD---TEQLKL
TaHsfA2a
TaHsfA2b
TaHsfA2c
TaHsfA2h
TaHsfA6a
TaHsfA6f
TaHsfA6e
TaHsfA8a
TaHsfA7a
TaHsfA7b
```

Supplementary Figure S2b. Amino acid sequence alignment of TaHsfA6f (GenBank accession #: KJ774108) with other full-length TaHsfA class members reported previously (Xue *et al*., 2014) **.**

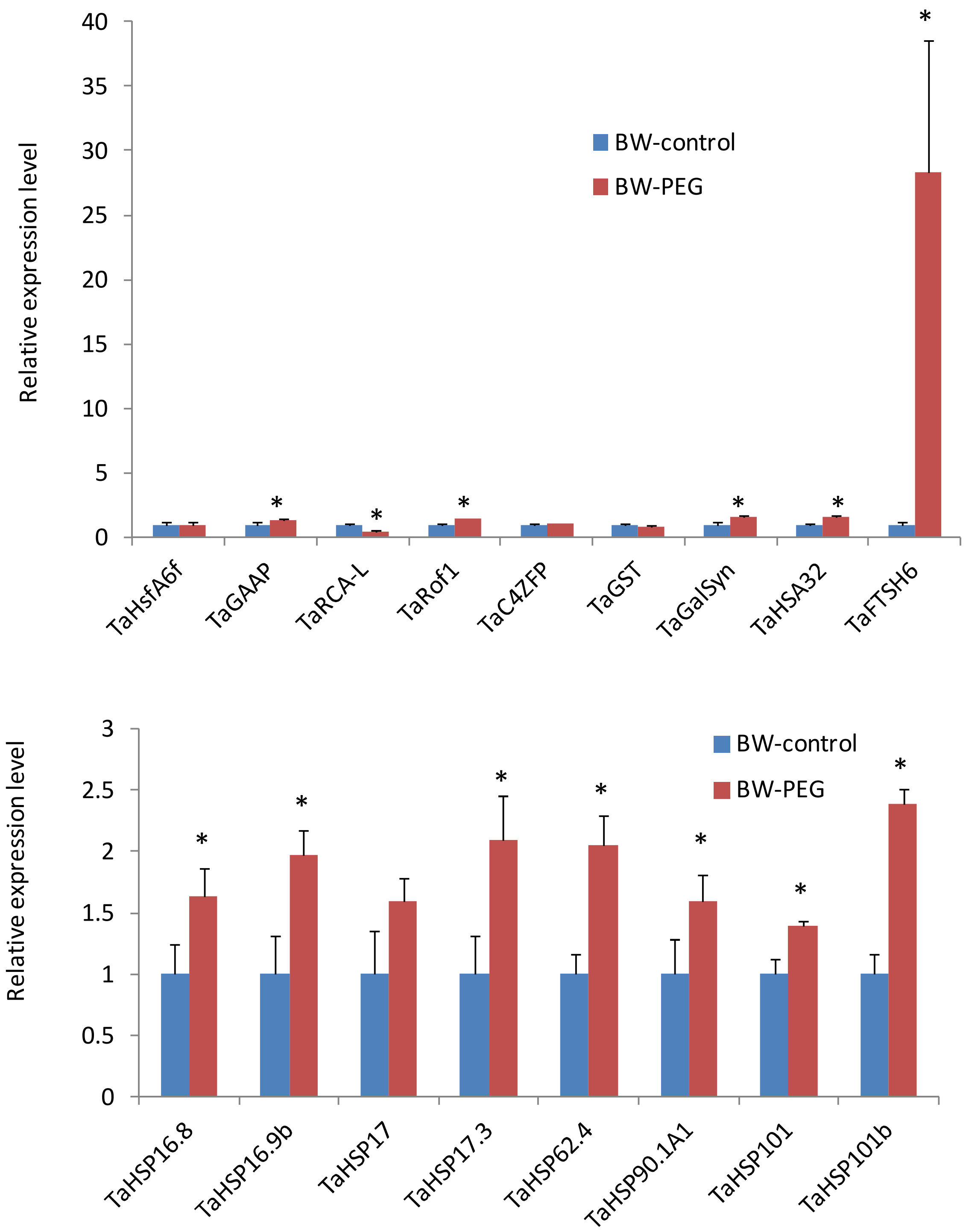

Supplementary Figure S3. Expression of *TaHsfA6f* and its target genes in wild-type plants in response to PEG treatment. Wheat [(cv. Bobwhite (BW)] seedlings (7-day-old) were treated with or without 15% of PEG for 3 days. Relative expression levels of genes were determined using quantitative RT-PCR. Values are means + SD of three biological replicates. Statistical significance (P < 0.05) of differences between control and PEG treated groups is indicated by an asterisk.

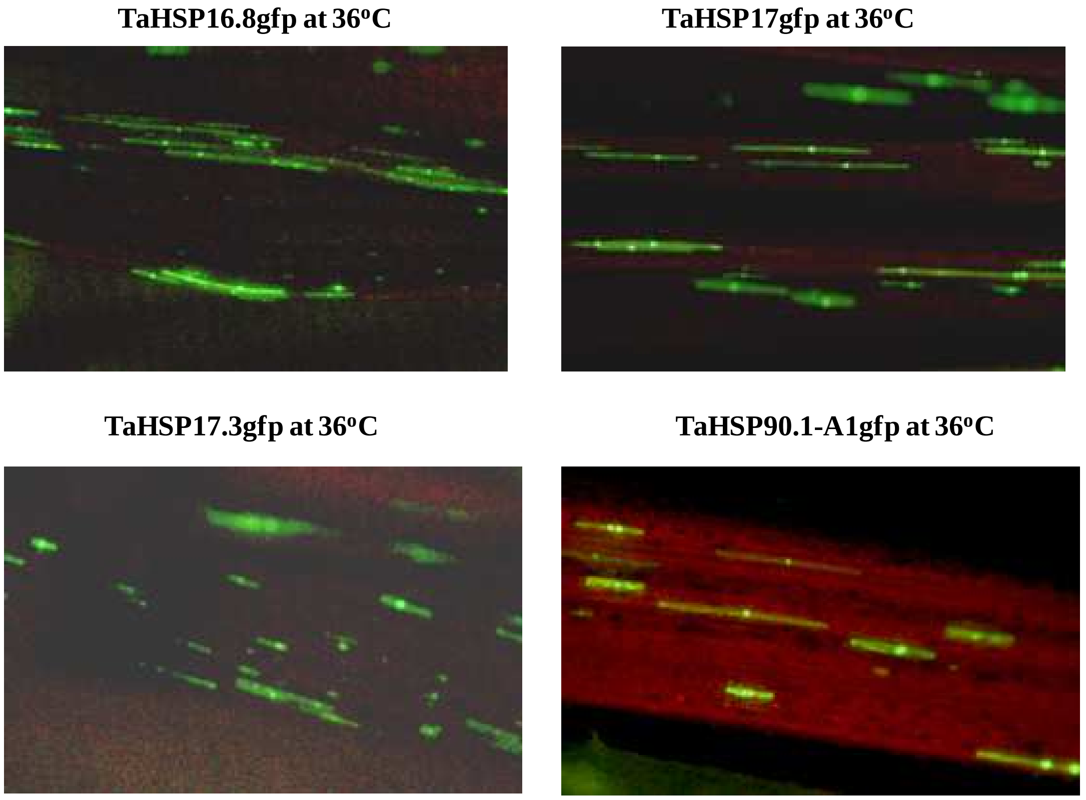

Supplementary Figure S4. The heat-induced expression of *GFP* reporter genes driven by HSP promoters. Expression of TaHSP promoter-driven reporter genes was analysed by bombarding a reporter construct into wheat seedlings. After bombardment, the seedlings were incubated at 36°C before examination of GFP foci.

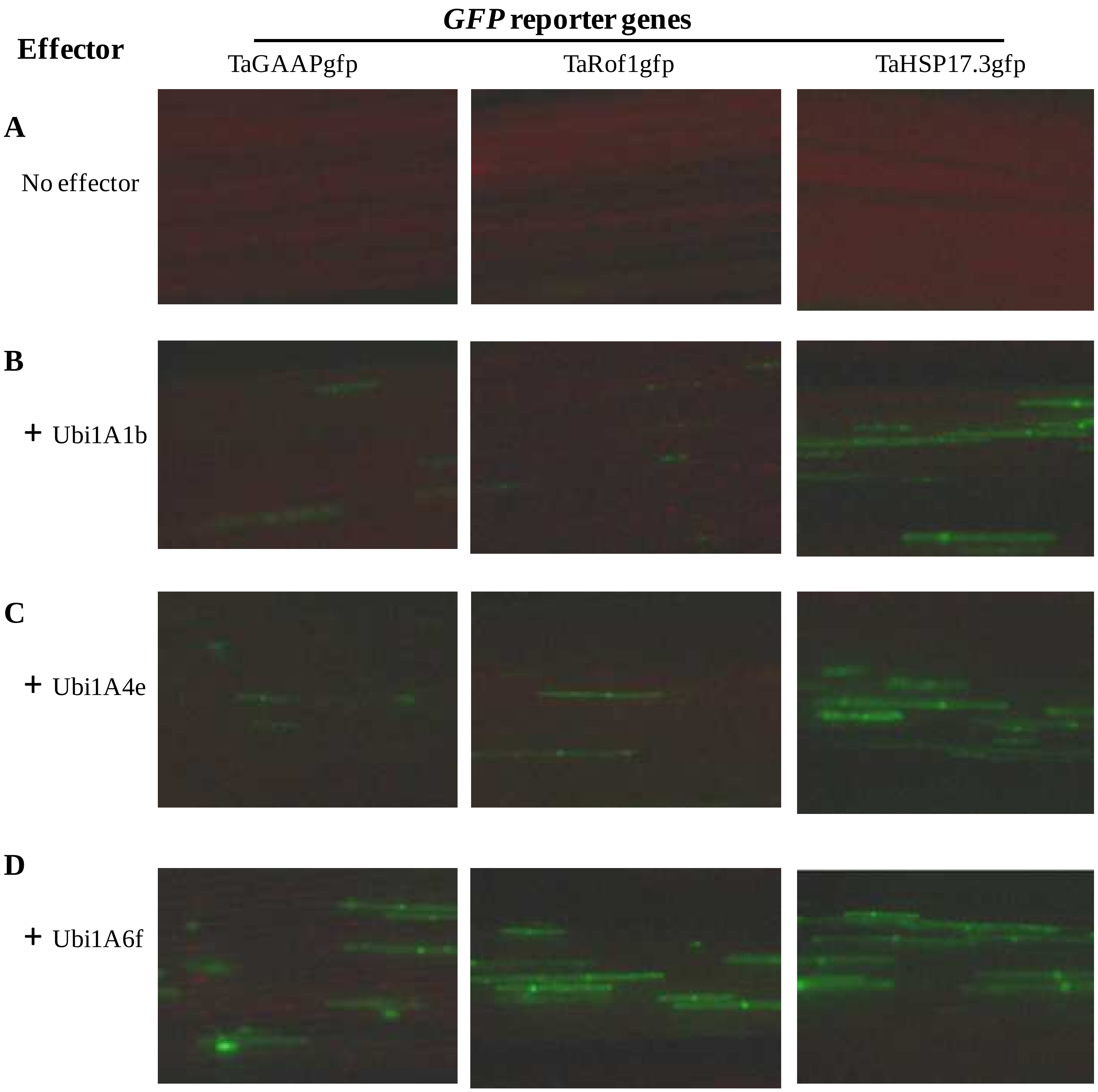

Supplementary Figure S5. Relative strength of TaHsfA6f transactivation activity in comparison with TaHsfA1b and TaHsfA4e. Three effector genes were under the control of a maize *Ubi1* promoter (Ubi1A1b, Ubi1A4e and Ubi1A6f). GFP reporter genes were under the control of a *TaGAAP*, *TaRof1* or *TaHSP17.3* promoter (TaGAAPgfp, TaRof1gfp and TaHSP17.3gfp). Each reporter gene was bombarded into wheat seedlings without an effector gene (**A**), or with an effector gene (**B**-**D**). After bombardment, samples were incubated at 22°C for 20 h before examining GFP foci. Each reporter and effector combination was analysed with four replicates. Representative sample sections for each combination are shown.

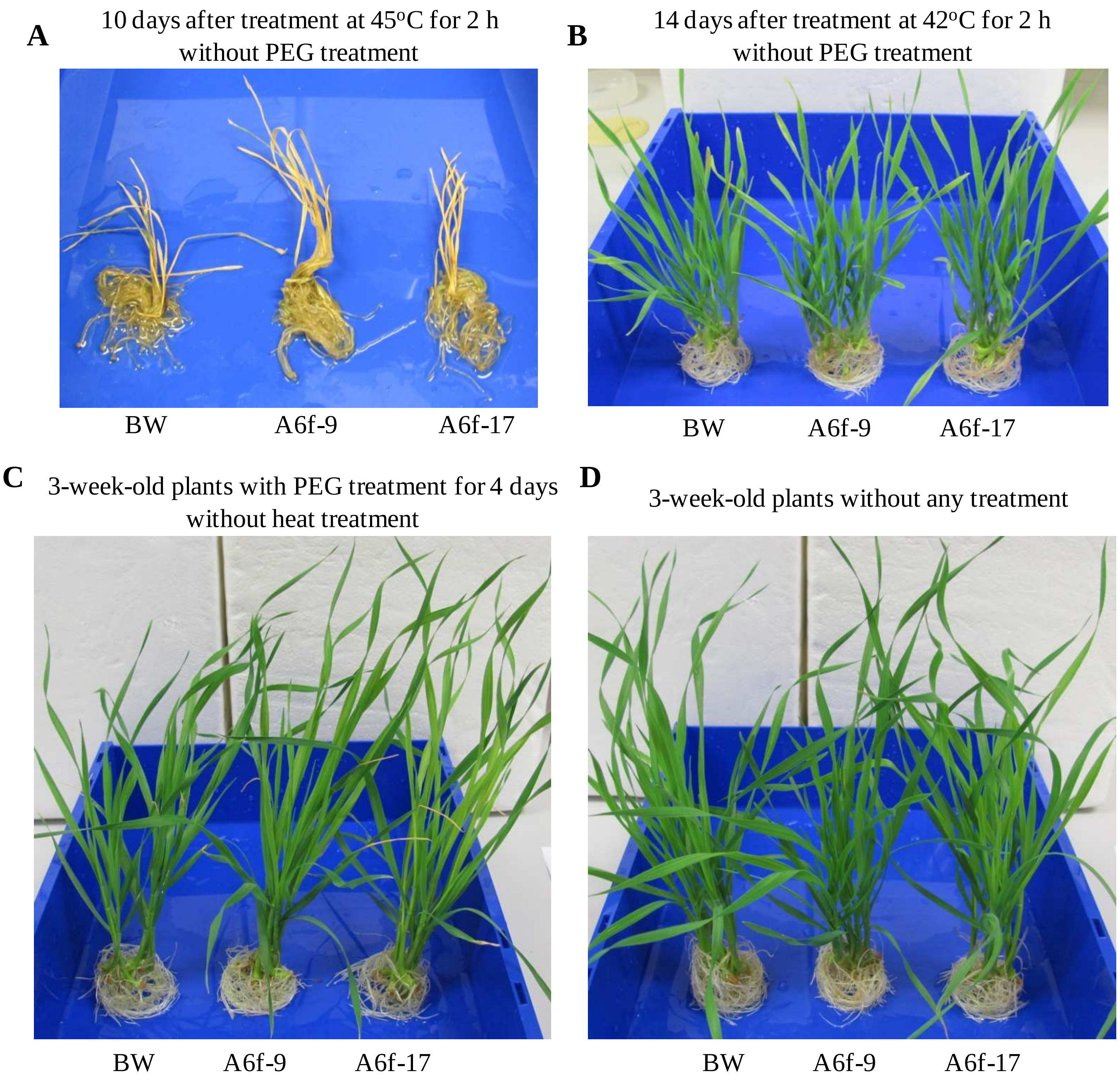

Supplementary Figure S6. Illustration of thermotolerance and growth of *TaHsfA6f* transgenic lines (A6f-9 and A6f-17) and Bobwhite (BW) under various control conditions. (**A**) Seven-day-old seedlings of BW, A6f-9 and A6f-17 were treated at 45°C for 2 h without PEG treatment. (**B**) Seven-day-old seedlings of BW, A6f-9 and A6f-17 were treated at 42°C for 2 h without PEG treatment. (**C**) Five-day-old seedlings of BW, A6f-9 and A6f-17 were treated with 15% PEG for 4 days. (**D**) Seedlings A6f-9, A6f-17 and BW without any treatment. Ten (**A** & **B**) or eight (**C** & **D**) seedlings of each genotype are shown. Seedlings were grown in a nutrient solution as described in the Materials and methods section. The mean ± SD values for shoot length (cm) of BW, A6f-9 and A6f-17 were 13.9 ± 1.5 , 14.0 ± 1.4 and 13.8 ± 1.4 in **B** (n = 30), 25.3 ± 2.1, 25.8 ± 2.3 and 25.7 ± 1.7 in **C** (n = 24), 26.1 ± 1.4, 25.9 ± 1.7 and 25.8 ± 1.6 in **D** (n = 24), respectively.

A Pre-treatment and post-treatment conditions for induction of *TaHSP17* promoter-driven *GFP* reporter expression and formation of GFP foci

	Genotype	Pre-treatment	Bombarded with construct(s)	Post-bombardment treatment	Formation of GFP foci
B1	Bobwhite	No	TaHSP17gfp	45°C for 2 h & then at 22°C	Not visible at any time
B2	Bobwhite	No	TaHSP17gfp	42°C for 2 h & then at 22°C	Not visible at any time
B3	Bobwhite	No	TaHSP17gfp	36°C for 4 h & then at 22°C	Start visible after 4 h
B4	A6f-17	15% PEG for 2 days	TaHSP17gfp	22°C	Start visible after 4 h
B5	Bobwhite	15% PEG for 2 days	TaHSP17gfp	22°C	Not visible at any time
B6	Bobwhite	No	TaHSP17gfp + HVA1sA6f	22°C	Start visible after 4 h

B

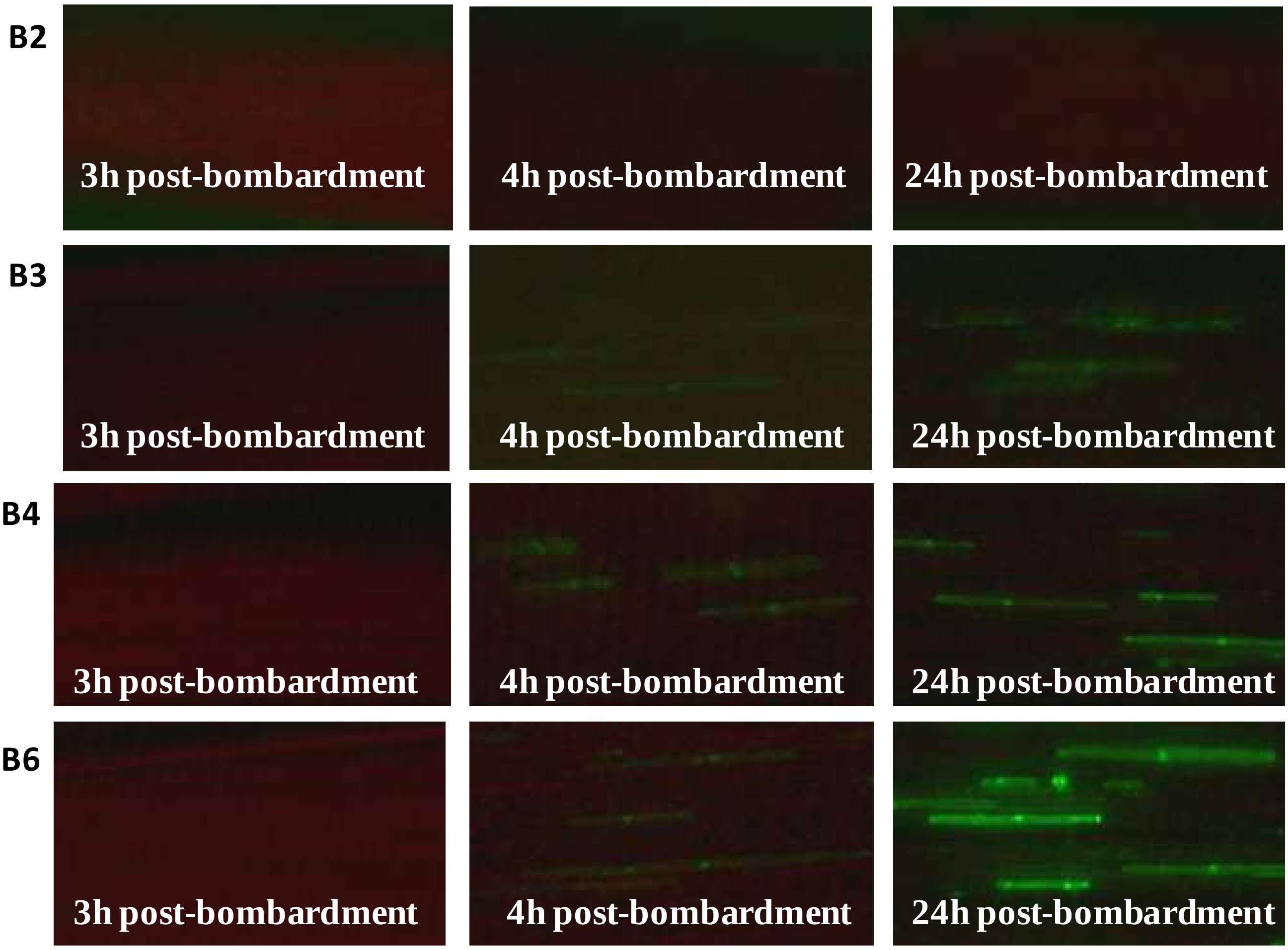

Supplementary Figure S7. Time course of the appearance of GFP foci produced by *TaHSP17* promoter-driven *GFP* reporter gene under various conditions. 1-week-old seedlings were placed on filter paper soaked with a solution of 50 mM sucrose, 15% PEG and 5 mM sodium phosphate buffer, pH 6.5 at least 1 h before bombardment and kept in this solution after bombardment. For the pre-treatment of B4 and B5 samples, five-day-old seedlings were grown in 15% PEG for 2 days. PEG was used to induce the expression of *HVA1s* promoter driven *TaHsfA6f* (HVA1sA6f). After bombardment, samples were immediately placed under post-bombardment conditions. Formation of GFP foci was monitored at 1-h, 2-h, 3-h, 4-h and 24-h post-bombardment. **A**, Treatment conditions**. B**, Illustration of GFP foci formation. GFP foci became detectable in B3, B4 and B6 at 4 h after bombardment. No GFP foci in samples [B1, B2 and B5 (data for B1 and B5 not shown)] at any time points. Much stronger GFP foci were seen in B6 than B4 at 24 h; this is because the copy number of the HVA1sA6f effector construct introduced into cells through the particle bombardment procedure in a transient expression system is usually much higher than that in the HVA1sA6f transgenic line through stably integration into chromosome.
